# One stone, many birds: Recent advances in functional nanogels for cancer nanotheranostics

**DOI:** 10.1002/wnan.1791

**Published:** 2022-03-25

**Authors:** Huiyi Wang, Matias L. Picchio, Marcelo Calderón

**Affiliations:** ^1^ POLYMAT, Applied Chemistry Department Faculty of Chemistry, University of the Basque Country, UPV/EHU Donostia‐San Sebastián Spain; ^2^ IKERBASQUE, Basque Foundation for Science Bilbao Spain

**Keywords:** bioimaging, magnetic resonance, nanogels, nanomedicine, theranostics

## Abstract

Inspired by the development of nanomedicine and nanotechnology, more and more possibilities in cancer theranostic have been provided in the last few years. Emerging therapeutic modalities like starvation therapy, chemodynamic therapy, and tumor oxygenation have been integrated with diagnosis, giving a plethora of theranostic nanoagents. Among all of them, nanogels (NGs) show superiority benefiting from their unique attributes: high stability, high water‐absorption, large specific surface area, mechanical strength, controlled responsiveness, and high encapsulation capacity. There have been a vast number of investigations supporting various NGs combining drug delivery and multiple bioimaging techniques, encompassing photothermal imaging, photoacoustic imaging, fluorescent imaging, ultrasound imaging, magnetic resonance imaging, and computed tomography. This review summarizes recent advances in functional NGs for theranostic nanomedicine and discusses the challenges and future perspectives of this fast‐growing field.

This article is categorized under:

Therapeutic Approaches and Drug Discovery > Emerging Technologies
Therapeutic Approaches and Drug Discovery > Nanomedicine for Oncologic Disease
Nanotechnology Approaches to Biology > Nanoscale Systems in Biology
Diagnostic Tools > In Vivo Nanodiagnostics and Imaging

## Graphical abstract

Nanogels are desirable candidates for cancer theranostic. As nanocarriers, their superior attributes endow them the ability to encapsulate therapeutics, and meanwhile generate diagnostic information under irradiation based on different techniques.

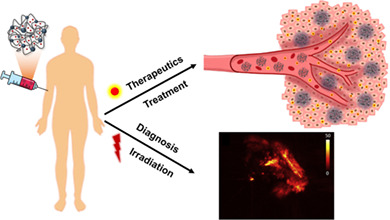

## 1 INTRODUCTION

**FIGURE 1:**
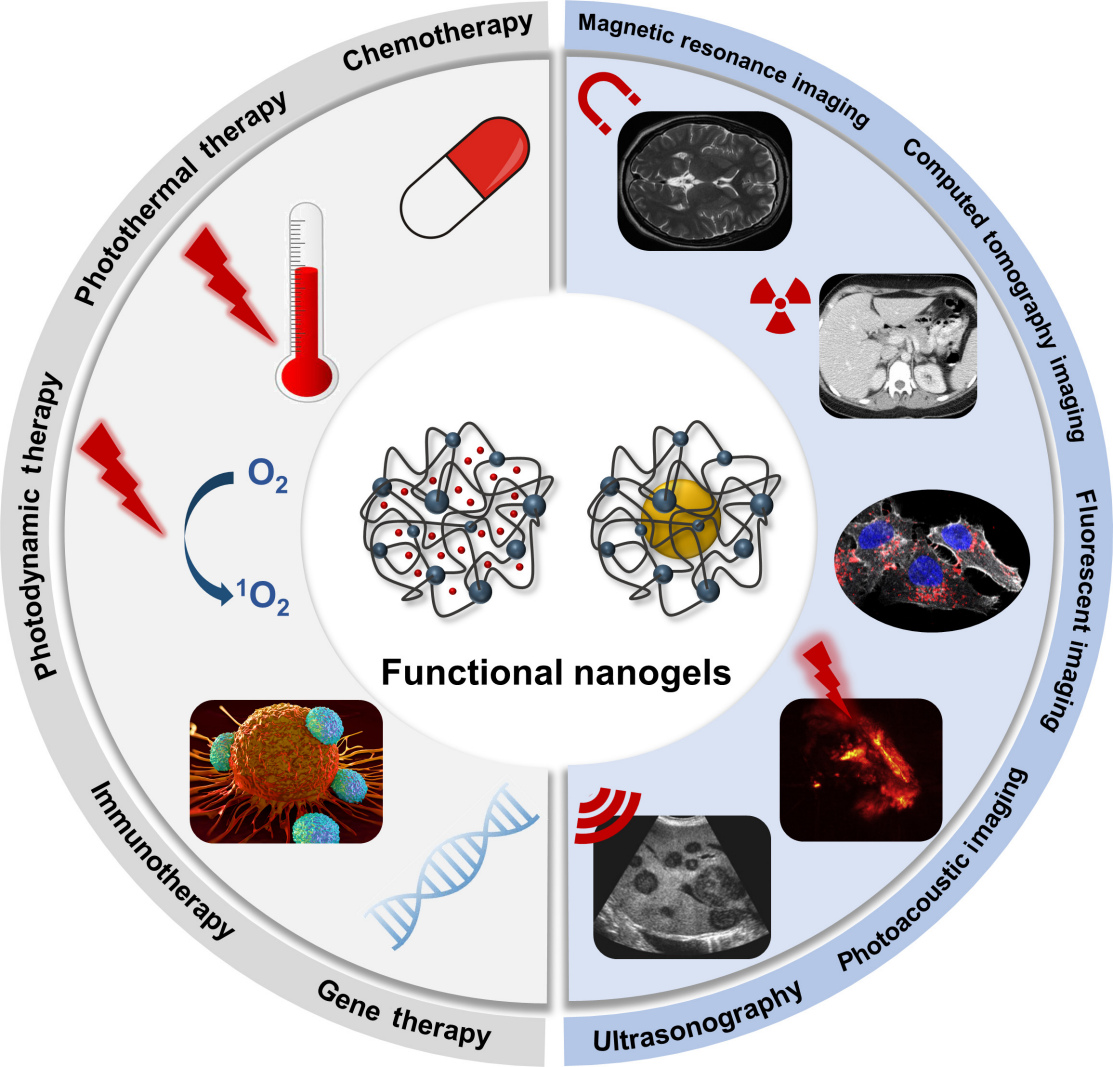
Current main tumor treatments and diagnostic techniques

In the 21st century, cancer is still one of the worldwide leading diseases with a high mortality rate (Sung et al., 2021). The urgent requirement for novel methodologies to diagnose and treat cancer has driven the rapid development of nanomedicine and nanotechnology. Even with certain limitations and some controversial viewpoints, nanotechnology does offer new hopes for efficient cancer therapy with higher accuracy and lower systemic toxicity than traditional treatments (Chen et al., 2017; Singh et al., 2020). Additionally, nanotechnology allows integrating therapy and diagnosis in one nanosystem, giving rise to the field known as nanotheranostics. Nanotheranostics is called to play a pivotal role in the early diagnosis of cancer, advanced drug delivery, imaging‐guided therapy, and posttreatment monitoring (Ang et al., 2021). Considering the diversity of cancer and individual patients, theranostic formulations should be pertinent and fit the state of the disease. Figure 1 shows the main tumor therapeutic and diagnostic approaches that are currently being studied. In terms of diagnosis, magnetic field, ultrasound waves, and various types of radiations have been commonly applied as the source for the existing imaging modalities, including magnetic resonance imaging (MRI), ultrasonography (US), computed tomography scan (CT), and positron emission tomography (PET). They have been demonstrated to be efficient clinical diagnostic techniques with high 2D/3D resolution. To increase the imaging quality, contrast agents (CAs) are recommended to be used, including small molecules and metal nanoparticles (Choi et al., 2014). For instance, gadolinium (Gd) (Robertson & Rendina, 2021) and iron oxide (Shell & Lawrence, 2015) nanoparticles (NPs) are conventional CAs for MRI. Instead, US typically utilizes microbubbles (Wilson & Burns, 2010). However, to obtain diagnostic information with higher precision and decrease the body's side effects from the radiation, light‐based diagnostic technologies, like fluorescent imaging (FLI), photoacoustic imaging (PAI), and photothermal imaging (PTI), appeared on the horizon to be the burgeoning research hotspots. Under the irradiation with near‐infrared (NIR) or ultraviolet–visible (UV–vis) light, the energy is transduced to generate pattern information.

Apart from diagnosis, light can also be the trigger valve for therapy, for example, in photodynamic therapy (PDT) and photothermal therapy (PTT) (Rai et al., 2010). PDT, which generates reactive singlet oxygen to elicit tumor cell death (Chen et al., 2020; Wang, Zu, et al., 2020), and PTT, which generates heat to kill tumor cells (Wei et al., 2019), are among the most promising approaches to perform precise treatment with lesser adverse effects than traditional chemotherapy. Although most of the ongoing research is still in the academic setting, scientists have started to translate the phototriggered theranostic from lab to clinical trials (Rai et al., 2010; Xu, Lu, & Lee, 2020). In addition, innovative approaches based on immunotherapy and gene therapy have been enthusiastically followed over the last decades as well (Dunbar et al., 2018; Zhang & Zhang, 2020). Immunotherapy aims to activate the immune cells (T cells, for instance) to recognize and attack cancer cells, establishing natural defenses and producing a long‐term and self‐sustained curing process (Schmidt, 2017). Moreover, gene therapy proposes to cure the disease via modifying the patient's genes, including replacing or inactivating the disease genes and introducing new/modified genes to boost the treatment (Bulaklak & Gersbach, 2020). Although gene therapy and immunotherapy are epoch‐making in nanomedicine for tumor treatment, there is still a long way to go before achieving wide‐scale application in the clinic due to the technical challenges and associated potential risks (Goswami et al., 2019; Hegde & Chen, 2020).

In order to carry out nanotheranostics with high efficacy, nanocarriers are utilized to deliver the therapeutics and imaging reagents to the desired target site, which brings tumor theranostics to a superior platform. Up to date, there have been numerous nanocarriers applied in tumor theranostics, like micelles, lipid nanoparticles, dendrimers, carbon nanotubes, hybrid nanoparticles, ionic liquid‐based nanoparticles, and nanogels (NGs) (Lim et al., 2015; Sangtani et al., 2017). Despite their proven performance, lipid nanoparticles and micelles show drawbacks regarding stability (He et al., 2019; Kim et al., 2010); while dendrimers and carbon nanotubes present long‐term nonspecific toxicity and low aqueous solubility (Li et al., 2019; Santos et al., 2020). Hence, eminent advantages from NGs have caught researchers' eyes (Sivaram et al., 2015; Wu & Wang, 2016). NGs are soft nanoparticles with a network of cross‐linked polymer chains showing high porosity. Due to their unique structure, they offer superior benefits over other nanocarriers, for example, high stability, flexibility and water content in aqueous dispersion, and good cellular uptake (Giulbudagian et al., 2018). Additionally, their high specific surface area contributes to a remarkable ability to encapsulate hydrophilic or hydrophobic payloads, including small therapeutic drugs, DNA/RNA sequences, imaging agents, and so on (Chacko et al., 2012; Molina et al., 2015). Based on the enormous arsenal of monomers for synthesizing NGs, multifunctional and highly biocompatible building blocks can be chosen to achieve high functionalization and low toxicity. Typically, NGs can be prepared from polymer precursors via self‐assembling and cross‐linking or monomers via homogeneous polymerization (Chacko et al., 2012). For example, Zhao et al. synthesized self‐assembled hybrid NGs consisting of Fe_3_O_4_ nanoparticles and Fmoc‐Tyr (H_2_PO_3_)‐OH polymers to achieve chemotherapy and ultrasound imaging (US) (Wu et al., 2021). Moreover, Calderon's group fabricated poly(*N*‐isopropylacrylamide) (PNIPAM)‐based NGs via precipitation polymerization for drug delivery devices with diagnosis capacities (Giulbudagian et al., 2016).

Furthermore, being sensitive to environmental stimuli helps NGs upgrade the importance in the research field of cancer nanotheranostics. Generally, non‐responsive NGs release payloads by diffusion in an uncontrolled manner. This property makes them more appealing to be conjugated with therapeutics or imaging agents via cleavable groups to achieve controlled release behavior. In addition, they have been widely used for the immobilization of photosensitizers or enzymes to maintain a proper concentration for efficient therapy (Qin et al., 2021). Nevertheless, responsive NGs undergo physiochemical changes responding to environmental stimuli, such as temperature, pH value, ionic strength, enzyme activity, radiation, and so on (Vicario‐De‐la‐torre & Forcada, 2017). Unlike typical nanocarriers, this property empowers the drug release in a controllable behavior, making it more achievable for topical and precise treatments on account of the differential conditions of tumor and physiological environments (Molina et al., 2015). Moreover, according to their composition, NGs are also classified into polymeric NGs and inorganic–organic hybrid NGs. On the one hand, as most of the CAs for bioimaging and sensitizers are of metallic nature, the effective combination of NGs and inorganic molecules can significantly improve theranostic efficacy (Sierra‐Martin & Fernandez‐Barbero, 2015; Zhang & Shi, 2020). On the other hand, organic molecules for bioimaging or as sensitizers gradually knock the door of tumor theranostic, addressing the side effects of the poor metabolism of the metal‐based compound inside the body (Chen et al., 2013; Fu, Yu, et al., 2020). In this review, we focus on the recent advances of functional NGs in cancer nanotheranostics. The fascinating properties of NGs allow them to be efficient nanocarriers for imaging agents and therapeutics. Herein, the design and application of NGs for different diagnostic modalities, including phototriggered imaging, MR, CT, and US imaging, will be discussed in detail.

## 2 NANOTHERANOSTIC BASED ON DIAGNOSTIC TECHNIQUES USED IN THE CLINIC

At present, MRI, CT, and US diagnostic modalities have shown significant predominance in clinical medicine owing to their valuable features, consisting of non‐invasiveness, fast acquisition time, and 3D imaging capacity of deep tissue and organs. In order to obtain images with good resolution, utilizing CAs is a key factor. Typically, traditional metal‐based CAs show poor cell uptake, tumor targeting, and biocompatibility when administered in a free form (Rogosnitzky & Branch, 2016). Hence, combining CAs with functional NGs and using metal‐free theranostic NG systems have drawn considerable attention. More recent developments consider as well the combination of NGs with therapeutics cargoes. This section summarizes the state‐of‐the‐art of theranostic NGs, involving hybrid as well as polymeric NGs, incorporating diagnostic techniques that are typically being used in the clinics. Table 1 shows some examples of functional NGs loading therapeutics and CAs for different treatments and imaging techniques.

**TABLE 1:** Functional NGs applied in theranostic using clinical imaging modalities

Nanogels composition	Imaging techniques	Contrast agents	Treatments	Therapeutics	Refs.
MOF@PEG	MRI	MnCo	PTT	PDA	Wang, Wu, et al. (2018)
PEI@Fe_3_O_4_	MRI	Fe_3_O_4_	Gene therapy	TFG‐β1 si RNA	Peng et al. (2021)
HA‐iodixanol	CT	Iodixanol	Chemotherapy	Paclitaxel	Zhu et al. (2016)
HA‐Cisplatin	CT	Cisplatin	Chemotherapy	DOX/cisplatin	Ma, Chen, et al. (2021)
MSN@DMAA	US	Perfluorohexane	Chemotherapy	DOX	Qiao et al. (2016)
MSN@Fmoc‐Tyr (H_2_PO_3_)‐OH	US	Catalase	Chemotherapy	DOX	Wu et al. (2021)
PEI‐FA‐PS	MRI/PAI	Gd/CuS	PTT	CuS	Zhang et al. (2020)
P(MAA/PEGMA)	PAI/FLI	CuS/Cy‐7	Chemotherapy/PTT	DOX/CuS	Zhang et al. (2019)

Abbreviations: DMAA, *N*,*N*‐dimethylacrylamide; HA, hyaluronic acid; MOF, metal–organic framework; MSN, mesoporous silica nanoparticles; PDA, polydopamine; PEG, poly(ethylene glycol); PEGMA, poly(ethylene glycol) methacrylate; PEI, poly(ethylenimine); PMAA, poly(methacrylic acid).

### 2.1 Magnetic resonance imaging

MRI technique, which started to be applied in clinics for body imaging in 1977, employs strong magnetic fields and radio waves to realign the protons from abundant hydrogen atoms in the body, generating detailed images of different tissues (Broadhouse, 2019). Based on the diagnostic requirements for different tissues, T_1_‐weighted and T_2_‐weighted images are the most common MRI basic pulse sequences, corresponding to spin–lattice relaxation and spin–spin relaxation, respectively. Among them, the T_1_‐weighted images highlight the fat content, while the T_2_‐weighted images highlight the water content of the body (Maeder et al., 2018). To collect radio signals of proton realignment from the magnetic field efficiently, metallic CAs are commonly employed for tumor MRI, such as Gd (Zhou & Lu, 2013), manganese oxide (MnO) (Na et al., 2007), Mn_3_O_4_ (Sun, Zhang, Zhang, Wang, et al., 2018)), iron oxide (Fe_3_O_4_) (Ma et al., 2020), and platinum (Pt) (Baklaushev et al., 2015).

Due to the adverse effects of conventional free‐form CAs, stemming from non‐biodegradability, accumulation in the kidney, and potential toxicity, NGs appear as good nanocarriers to enhance their performance and boost their therapeutic functions. Owing to their stability in aqueous dispersion and high water content, NGs give CAs better adaptiveness for MRI (Lux & Sherry, 2018). Besides, the introduction of NGs enhances the relaxivity and extends the residency of the CAs in blood circulation, which is owing to the retarded rotational motion and enhanced size after the conjugation between CAs and polymers, respectively (Soleimani et al., 2013; Tang et al., 2013). Gd and Mn, as the most typical T_1_‐weighted positive CAs, have attracted much attention (Xu et al., 2018; Zhou & Lu, 2013). Shi et al. fabricated thermo‐responsive and acid‐degradable poly(*N*‐vinylcaprolactam) (PVCL) NGs with lower critical solution temperature to load Gd‐Dotarem chelates for in vivo T_1_‐weighted MRI (Sun et al., 2017). This hybrid NGs system showed in‐vivo tumor diagnostic feasibility with higher *r*_1_ relaxivities (*r*_1_ = 1/T_1_, 6.38–7.10/mM/s) and imaging intensity than the current clinical probes. They can be explained as the conjugation with NGs slowed down the rotational motion corresponding to the proton relaxation process and increased the concentration of Gd chelates in the blood. Besides, by the encapsulation in NGs, Gd‐Dotarem chelates acquired longer blood circulation time resulting in longer imaging time.

**FIGURE 2:**
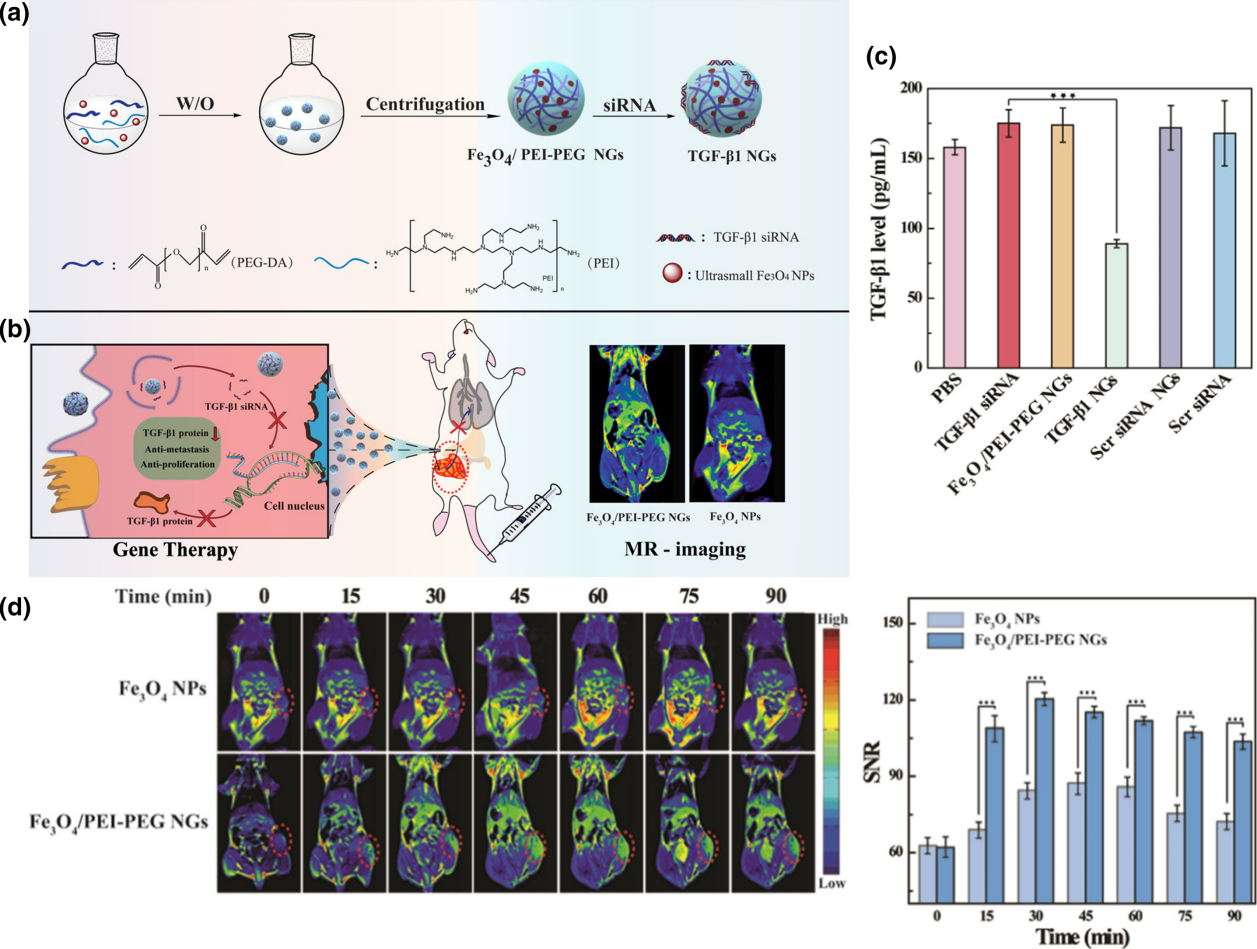
(a) Schematic representation of the synthesis of Fe_3_O_4_‐encapsulated PEI‐PEG NGs and (b) the mechanism for tumor theranostic. (c) Intracellular TGF‐β1 levels of PBS, free TGF‐β1, Fe_3_O_4_/PEI‐PEG NGs, TGF‐β1 NGs, scrambled (Scr) siRNA NGs, and Scr siRNA incubated cells, which were determined by the ELISA kit assay. (d) In vivo T_1_‐weighted MRI and the corresponding signal‐to‐noise ratio (SNR) value of xenografted S180 tumors at different time points with the intravenous injection of Fe_3_O_4_ NPs and Fe_3_O_4_‐encapsulated PEI‐PEG NGs. Reprinted with permission from Peng et al. (2021). Copyright 2021. American Chemical Society

Apart from Gd chelates, superparamagnetic ultrasmall Fe_3_O_4_ NPs also exhibit good biocompatibility and flexibility, and higher *r*_1_ relaxivity (Thapa et al., 2017). The size of Fe_3_O_4_ NPs determines the weighted sequence: Fe_3_O_4_ NPs of diameter more than 5 nm are better fitted for T_2_‐weighted MRI with enhanced dark signal contrast, while those smaller than 5 nm are more suitable for T_1_‐weighted MRI with brightening signal (Ma et al., 2020). In an attempt to combine T_1_‐weighted MRI and gene therapy, PEI–PEG NGs were formed with a size of 76.3 ± 15.61 nm and loaded with ultrasmall Fe_3_O_4_ NPs, which were synthesized by solvothermal route, as shown in Figure 2a,b (Peng et al., 2021). In this study, taking into account the tolerability and the efficiency as gene vector, low‐molecular‐weight PEI was cross‐linked with PEG via inverse microemulsion approach, reaching low toxicity and admirable gene delivery capacity (Figure 2c). The hybrid NGs presented higher *r*_1_ relaxivity of 1.0346 mM^‐1^·s^‐1^, with higher quality and prolonged performance (even up to 90 min) of in vivo MRI than solo ultrasmall Fe_3_O_4_ NPs (Figure 2d). It was demonstrated that NGs moiety promoted the absorption of the CAs in tumor cells and prolonged their active time due to their intrinsic flexibility and positively charged surface. As an anti‐metastatic approach, TGF‐β1 siRNA was engaged to silence the growth factor TGF‐β1. In vivo tumor inhibition assay proved that NGs effectively protected siRNA and knocked down the expression of TGF‐β1, showing an impressive reduction of migration and promotion of apoptosis of sarcoma tumor cells. Nonetheless, superparamagnetic Fe_3_O_4_ NPs still possess some drawbacks, such as negative contrast effects and magnetic susceptibility artifact of T_2_ MRI, which need to be further studied (Na et al., 2007).

**FIGURE 3:**
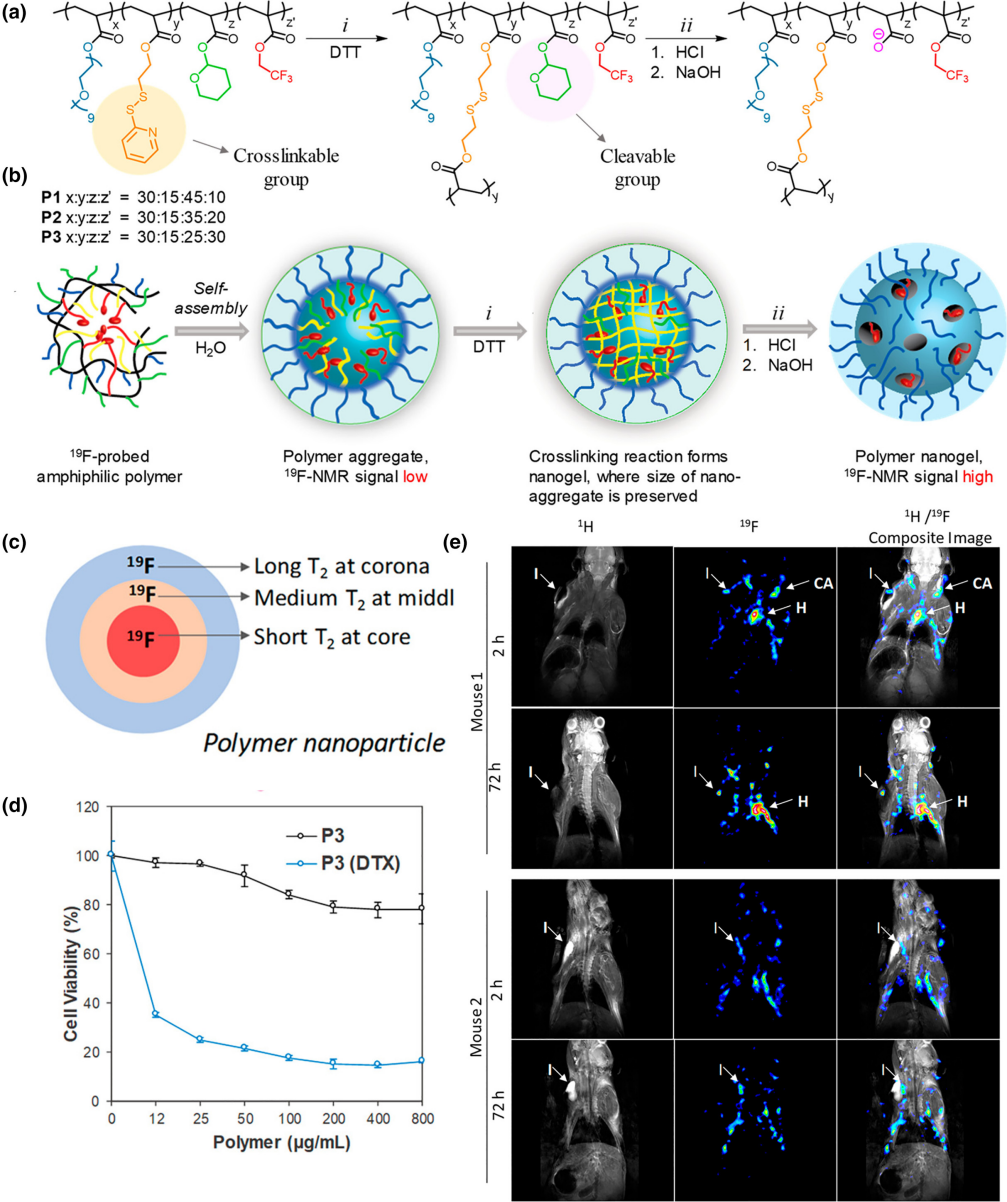
(a) Synthesized methodology and (b) schematic representation of the ^19^F‐fluorinated self‐assembly NGs with decreased interior density. (c) Variate relaxation of T_2_ in the different layers of the NGs depending on the fluorine microenvironment. (d) Cell viability with NG and NG‐DTX incubated under different concentrations. (e) In vivo MRI at the inflammation site over 72 h. Reprinted with permission from Munkhbat et al. (2019). Copyright 2019. American Chemical Society

However, the limited sensitivity of ^1^H MRI requires a high concentration of CAs. Furthermore, the conventional metallic CAs have a long‐term accumulation in the body, which poses potential safety problems (Tirotta et al., 2015). In comparison, metal‐free organic CAs display significant superiority, such as functionality, relatively low toxicity, improved pharmacokinetics, and favorable biodistribution (Akakuru et al., 2019). According to different contrast mechanisms, they could be divided into organic radical, chemical exchange saturation transfer, and heteronuclear CAs (Fu, Yu, et al., 2020). For instance, ^19^F, as a heteronuclear atom, has been widely studied to overcome the limitations of the traditional ^1^H MRI technique, due to the considerably high MR sensitivity (83% of the ^1^H protons) without background signal from the body, a broad range of chemical shift (>350 ppm), 100% natural abundance and nuclear spin of 1/2 (Ahrens & Bulte, 2013; Dolbier, 2008; Tirotta et al., 2015; Wolters et al., 2013). Besides, the gyromagnetic ratio of ^19^F (40.078 MHz/T) is quite close to hydrogen (42.577 MHz/T), revealing their similar imaging capacities. To ensure the high sensitivity of ^19^F MRI, the fluorine content should be high enough in the NGs. Nevertheless, the hydrophobic fluorocarbon segments induce aggregation. Therefore, the design of NGs with appropriate fluorine is a challenge for metal‐free NGs theranostic platforms. Resourcefully, Thayumanavan et al. took advantage of the hydrophobic fluorocarbon segments to fabricate self‐assembled NGs via the chemical cross‐linking of ^19^F‐probed amphiphilic copolymers (Figure 3a; Munkhbat et al., 2019). In order to decrease the loss of NMR and relaxation time, the fluorine content and segmental mobility in the hydrophobic ^19^F‐probed core should be sufficient. Hence, tetrahydropyranyl, which can be degraded by acid, was applied to form the interlayer of the NPs for generating the loose interior (Figure 3b). In this case, differential T_1_ relaxation was observed in different parts of the polymeric nanoparticles, resulting from an inhomogeneous distribution of ^19^F concentration (Figure 3c). Figure 3d illustrates that docetaxel‐encapsulated NGs exhibit satisfactory cell killing ability at 10 μg/ml, whereas they do not show apparent cellular toxicity. Afterwards, the in vivo imaging assay indicated that the prepared ^19^F‐probed NGs had a long blood circulation and accumulated around the inflammation site, as shown in Figure 3e. Besides, the as‐prepared NGs were decorated with folic acid, showing efficient receptor‐mediated cellular uptake. Overall, these NGs incorporating metal‐free CAs are good candidates for delivering therapeutics along with MRI diagnosis. Whereas, ^19^F MRI technique and organic CAs are limited in the clinic for various reasons, like high costs and rapid clearance, which requires further improvement.

### 2.2 Computed tomography scan imaging

Similar to the time of MRI development, CT scan emerged in the 1970s. During the CT scanning process, X‐ray is utilized as irradiation to create images based on the signal of X‐ray attenuations. Compared with the MRI technique, CT scan possesses a higher spatial resolution and contrast. As for the CAs, iodine molecules, lanthanide molecules, gold nanoparticles (Zhu et al., 2017), bismuth molecules (Fu, Guo, et al., 2020), and platinum (Ma, Chen, et al., 2021) are commonly used (Lusic & Grinstaff, 2013).

**FIGURE 4:**
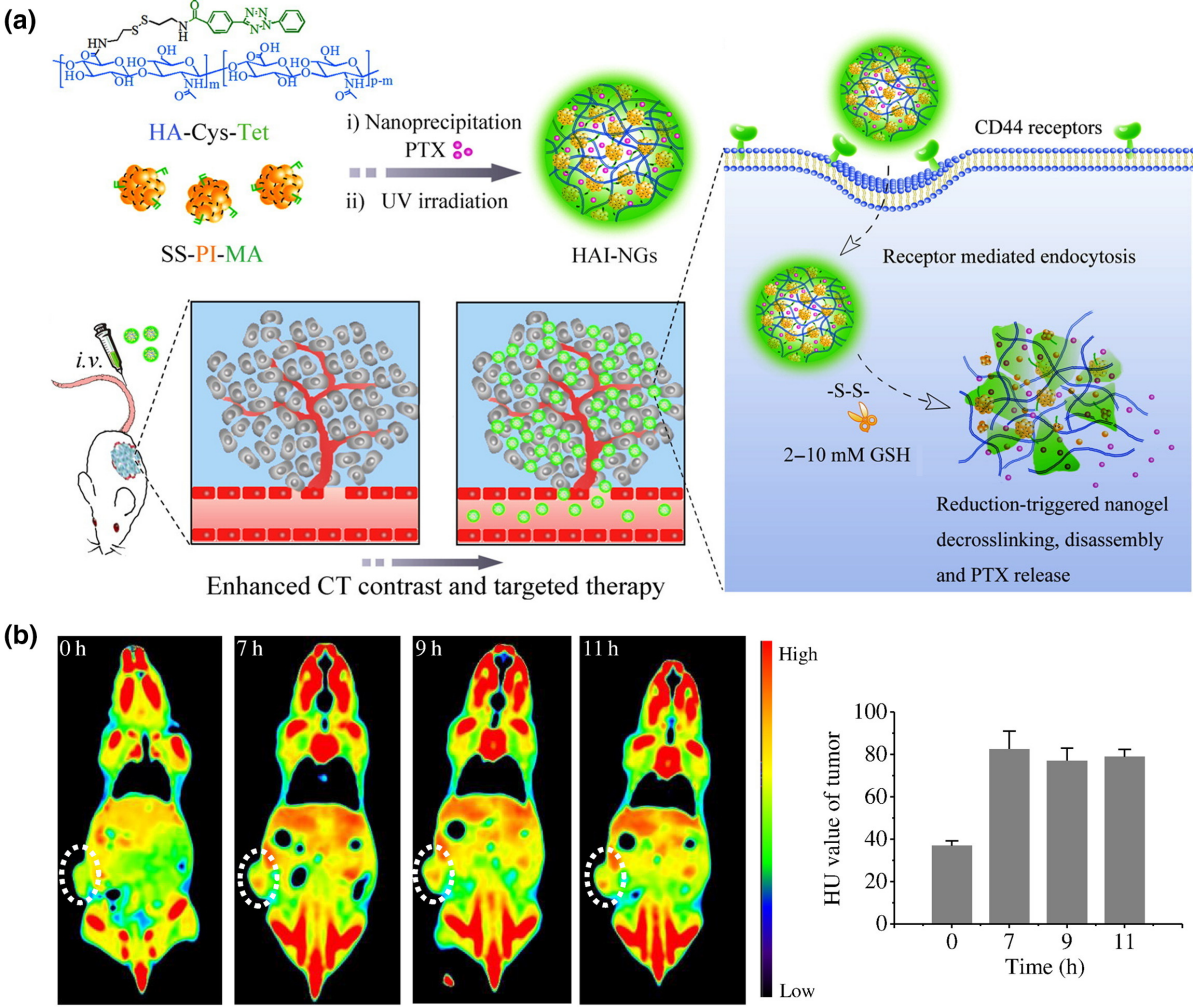
(a) Illustration of bioresponsive and fluorescent hyaluronic acid‐iodixanol NGs for targeted X‐ray CT imaging and chemotherapy of breast tumors. (b) Axial CT images and the corresponding HU value of MCF‐7 tumor‐bearing mice at 0, 7, 9, and 11 h following tail vein injection of HAI‐NGs at a concentration of 15 mg/ml (60 mg iodine equiv./kg). Circled areas indicate the position of the tumor. Reprinted with permission from Zhu et al. (2016). Copyright 2016. Elsevier

To enhance the stability and imaging performance of CAs, NGs have been shown to count with ideal properties (Sun, Zhang, Zhang, Zhou, et al., 2018). For instance, for tumor imaging, the mechanical properties of NGs can promote the accumulation and penetration of CAs in the tumoral tissue (Li et al., 2020). As an example, Zhong et al. formulated hyaluronic acid‐iodixanol NGs (HAI‐NGs) via nanoprecipitation and photoclick cross‐linking reaction for targeted chemotherapy and CT imaging, as shown in Figure 4a (Zhu et al., 2016). In this NG system, glutathione (GSH)‐degradable polyiodixanol‐methacrylate was employed for controllable drug release and CT imaging. It is known that CD44 is a type I transmembrane glycoprotein showing good binding to hyaluronic acid (HA) (Misra et al., 2015). Toward the overexpression of CD44 receptor in tumor cells, the utilization of HA endows the system with targeting drug delivery and imaging capacity, which is essential for tumor theranostic. Figure 4b reflected that HAI‐NGs can target and accumulate in MCF‐7 tumor, showing an enhanced Hounsfield units (HU) value from 30.7 to 82.6 after 7‐h intravenous injection of HAI‐NGs. This is advantageous for clinical application. In addition, it was worth noting that based on the in vivo pharmacokinetics investigation, paclitaxel displayed a prolonged blood circulation after being encapsulated into NGs, even with a detectable level after 24 h. This was owing to the interaction between paclitaxel and the functional groups in the NGs.

Similarly, in another study, self‐targeting hyaluronate NGs (HANG) were synthesized using cisplatin (CDDP) as a cross‐linker for loading doxorubicin (DOX) and performing synergistic chemotherapy and CT imaging (Ma, Chen, et al., 2021). Cisplatin is a multifunctional drug commonly used in cancer therapy and CT imaging (Schwartz et al., 2011). In this theranostic system, DOX and cisplatin exhibited pH‐independent release, along with synchronized and decelerated pharmacokinetics. After 6 h of injection in mice bearing MCF‐7/ADR tumor, a clear tumor signal was observed from CT image, which got an enhancement of contrast after 12 h only at the tumor position. This study and others demonstrated that theranostic NG systems could improve the circulation time of CAs, enhance imaging quality, and present controlled drug release behavior. Despite such promising results, conventional CAs still possess some drawbacks. For instance, iodine‐based molecules and cisplatin accumulate in the body generating severe renal toxicity (Davenport et al., 2020; Hanigan & Devarajan, 2003). As alternatives, gold, bismuth, and lanthanide nanoparticles have piqued much attention. For example, the application of bismuth can reduce the radiation dosage on radiosensitive tissue position (Liao et al., 2019). Lanthanide nanoparticles show lower biotoxicity and higher contrast signal compared to heavy metals (Prodi et al., 2015). Besides, Au nanoparticles possess good biocompatibility, exhibit suitable X‐ray attenuation properties (Dong et al., 2019) and have ideal properties as photothermal agents for PTT (Vines et al., 2019). Shi et al. designed γ‐polyglutamic acid (γ‐PGA) NGs with Au NPs loaded via a double emulsion approach (Zhu et al., 2017). After the encapsulation of Au NPs into NGs, dramatically enhanced cellular uptake of Au was observed by means of inductively coupled plasma optical emission spectroscopy (ICP‐OES), especially with an Au concentration of 0.4 mM. In vivo CT imaging study showed that the CT signal in the tumor position started immediately after injection, reaching a maximum in 2 h. The evaluation by ICP‐OES demonstrated that the Au NPs‐loaded NGs start to be eliminated from the body after 2 h. It was concluded that Au NPs‐loaded NGs could accumulate in the tumor through the passive enhanced permeability and retention effect (EPR) and show better biocompatible than other conventional imaging systems owing to the clearance process.

Even though CT imaging technique is reliable and has been applied in clinics for a long time with outstanding imaging capacity and depth, it still carries some drawbacks. X‐ray and radio‐CAs may bring adverse effects to the body, like damage to the healthy cells and itching rash for the skin, which put an obstacle on developing CT techniques in nanomedicine and nanotechnology.

### 2.3 Ultrasound imaging

Over the last 20 years, US imaging, also called ultrasonography, has been one of the most widely applied clinical non‐invasive imaging modalities to explore fetuses, bone, abdomen, breast, and so on (Carovac et al., 2011). Different from the radiation of CT, US imaging uses high‐frequency sound waves and produces real‐time images based on the wave reflection on the body structure. For example, the blood flowing in the blood vessels and the fetal situation can be monitored through US imaging.

**FIGURE 5:**
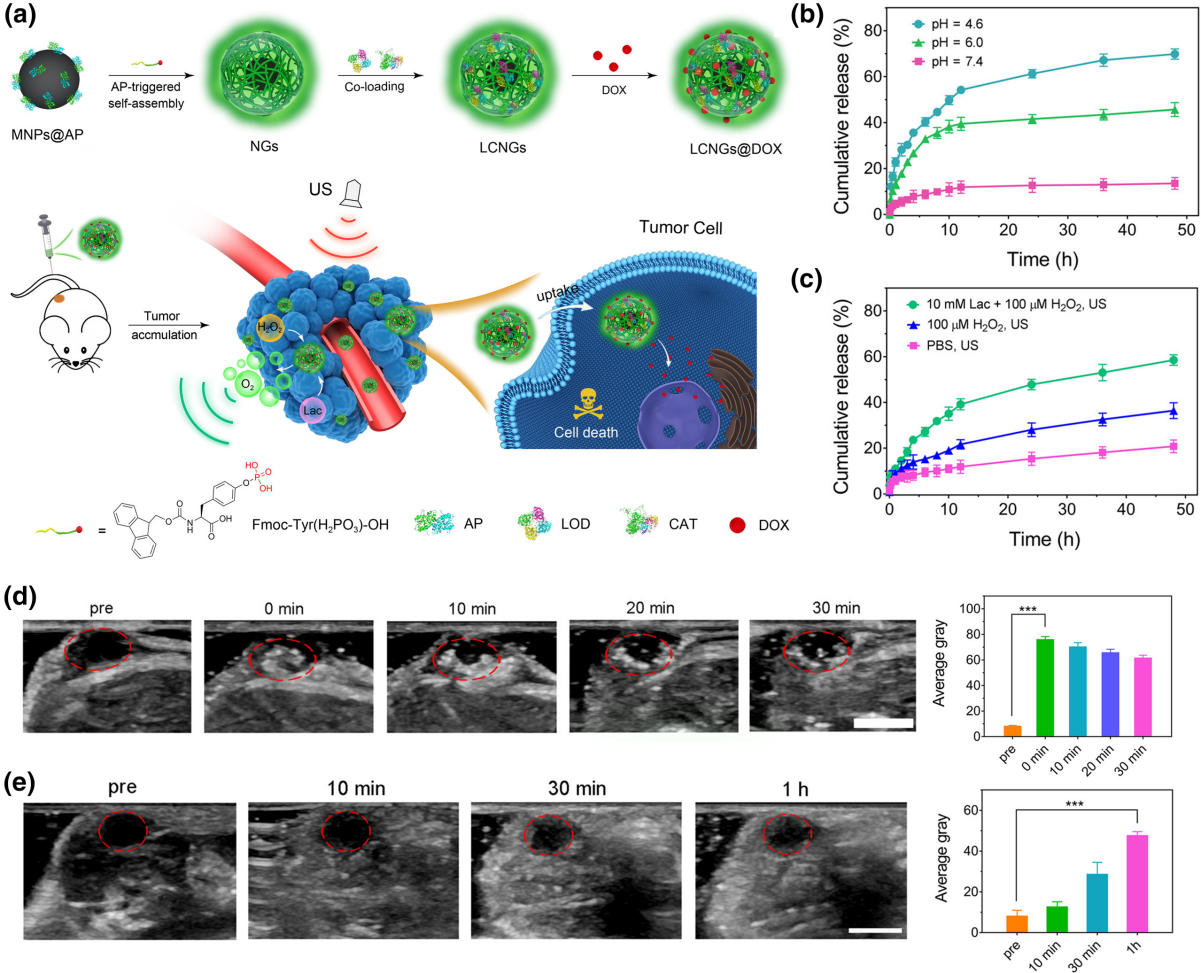
(a) Schematic illustration of self‐assembled hybrid NGs for enhanced enzyme‐regulated US and tumor chemotherapy. DOX release profile from LCNGs under varied pH values (b) and substrates by the irradiation of ultrasound (c) at 37 °C. (d) In vivo tumor US images and their corresponding mean gray values of the mice before and after intra‐tumorous injection of LCNGs under different time points (pre, 0, 10, 20, and 30 min). Circles in redlines indicate the injection sites in the tumors. Scale bar is 0.5 cm. (e) In vivo US images and their corresponding mean gray values of the tumor‐bearing mice before and after intravenous injection of LCNGs at different time points (10, 30, 60 min). Reprinted with permission from Wu et al. (2021). Copyright 2021. American Chemical Society

O_2_ content in the tissue is an important factor for transmitting US waves to yield images with good quality. However, hypoxia is a feature of the tumor microenvironment (TME), which might affect the quality of tumor US imaging. Then, gas‐filling microbubbles are commonly used to improve the acoustic environment of TME (Zhou et al., 2013). However, the weak subharmonic response at high‐frequency waves and poor stability of microbubbles limit their development in tumor US imaging, resulting from the microscale size distribution and diffusion of the core gas, respectively (Lee et al., 2017). By means of the excess of hydrogen peroxide (H_2_O_2_) in TME, nanosized catalase (CAT) is an excellent candidate to increase the concentration of O_2_ for further enhanced US imaging intensity through decomposition reaction of H_2_O_2_, which generates H_2_O and O_2_. Besides, lactate oxidase (LOD) enables to enhance the H_2_O_2_ level in the TME by reacting with lactate. It was hypothesized that the combination of CAT and LOD could significantly boost the accumulation of O_2_ bubbles in the TME via two reactions in cascade. To prove this, NGs have been used as enzymatic carriers, enhancing their stability in physiological media and their TME targeting ability. Self‐assembled multifunctional hybrid NGs (LCNGs) with dual enzymes (CAT and LOD) and loaded with DOX·HCl were fabricated by Zhao et al., as shown in Figure 5a (Wu et al., 2021). In this system, acid phosphatase (AP)‐modified magnetic nanoparticles (MNP) were chosen to be the template to trigger the self‐assembled formation of NGs shell on the surface of MNP by means of Fmoc‐Tyr (H_2_PO_3_)‐OH. The high amount of carboxyl groups inside the NGs promoted DOX·HCl encapsulation via electrostatic interactions and boosted the DOX release in an acidic environment. Moreover, the addition of LOD increased the H_2_O_2_ amount, while CAT enhanced the concentration of generated O_2_ in the TME. In vitro DOX release profiles revealed enhanced release under a lower‐pH environment (Figure 5b). Besides, the generation of O_2_ accelerated the DOX release as well (Figure 5c). It was verified that the US imaging signal was dramatically enhanced under the lactate + H_2_O_2_ environment in vivo with both intratumorous (Figure 5d) and intravenous (Figure 5e) injection, showing an increased average gray value. Similarly, Hest et al. formulated pH‐cleavable PEGylated NGs with single‐enzyme loaded for ultrasound wave‐guided chemotherapy and focused ultrasound ablation (Zhu, Li, et al., 2019). Thanks to the protection of NGs shell, the CAT enzyme was prevented from being dissociated under focused ultrasound and got prolonged in vivo circulation time while significantly improving the US imaging. Interestingly, the introduction of the focused ultrasound technique intensified the DOX treatment and optimized the TME for US imaging.

To accumulate gas bubbles in TME, small perfluorocarbon molecules are another popular category of US CAs. The perfluorocarbons with low boiling points, like perfluorohexan, can evaporate at physiological temperature spontaneously to generate gas for enhancing US imaging. In order to load DOX and perfluorohexan for chemotherapy and US imaging‐guided high‐intensity focused ultrasound, Wang et al. prepared mesoporous silica nanoparticle‐based ternary inorganic supramolecular polymeric NGs (MSN‐GII) (Wang et al., 2019). The peptide gelators, NapFFK and NapFFK‐acrylic, were self‐assembled on the surface of activated MSN by enzymatic polymerization and disulfide‐mediated cross‐linking. The hybrid system allowed to simultaneously encapsulate the hydrophobic molecule, perfluorohexan, and the hydrophilic molecules, DOX·HCl. This system showed a robust drug release under the simulated enzymatic and reducing TME (Dithiothreitol + Proteinase K). Moreover, in vitro and in vivo assays demonstrated that the multi‐functional hybrid NGs possessed superior US imaging capacity by the feat of elastic NG shell.

Overall, functional NG systems have achieved significant development in cancer nanotheranostic with the aid of clinical imaging techniques. The pharmacokinetics and biodistribution of CAs can be improved by the conjugation with NGs, exhibiting enhanced imaging efficiency and decreased systemic toxicity. However, most of current functional NGs for MRI, CT, and US imaging are still in the academic stage. The challenges of dose requirement, long‐term toxicity, and in vivo background signal leave them a big development space for the transition from academy to the clinic.

## 3 NANOTHERANOSTICS BASED ON UV–VIS LIGHT AS TRIGGER

**FIGURE 6:**
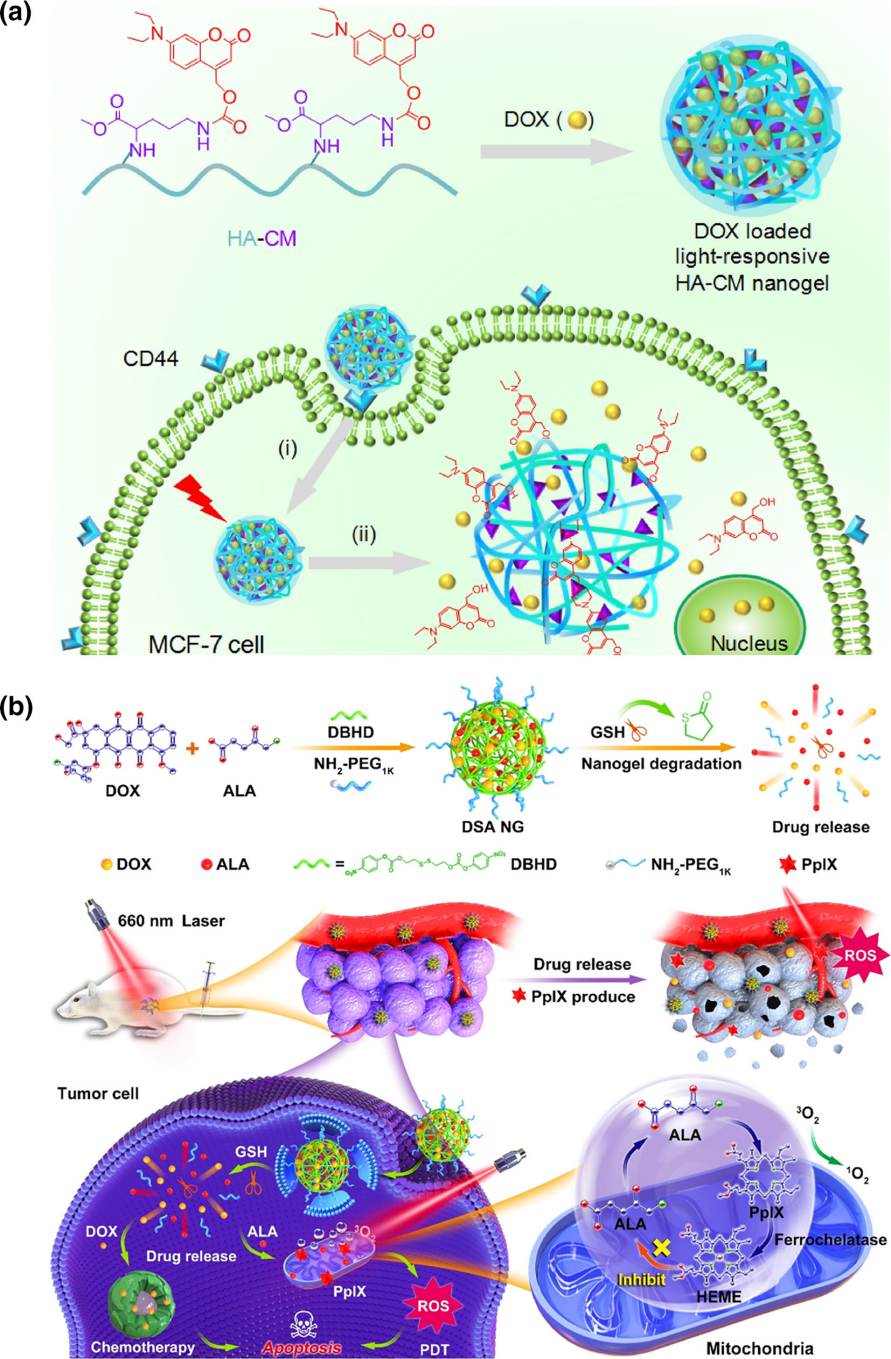
(a) Light‐responsive HA NGs for remotely controlled DOX delivery. Reprinted with permission from Hang et al. (2017). Copyright 2017. Elsevier. (b) Schematic illustration of the working mechanism of DSA NG; nuclear and mitochondria drug delivery could be achieved. Reprinted with permission from Wang, Zu, et al. (2020). Copyright 2020. American Chemical Society

Even though clinical imaging modalities have shown remarkable advances in spatiotemporal accuracy, like emerging 4D imaging tools, they still have some shortcomings regarding synergistic therapy, whereupon phototriggered theranostic steps into the spotlight. The use of light as a remote‐activation mechanism for triggering therapeutic effects and enhancing diagnosis is one of the most popular tools in cancer theranostic (Ai et al., 2016; Han & Xie, 2016). The easy manipulation offered by light allows accurate cancer targeting, low invasiveness, precise spatiotemporal control of payload release, and novel mechanisms of action (Khan et al., 2021; Shi & Sadler, 2020). Hence, phototriggered theranostic nanoparticles make it possible to visualize and treat tumors in a controlled fashion with minimal side effects (Abueva, 2021). Several innovative and robust light‐sensitive theranostic NGs have been developed over the last decade based on organic dyes, semiconducting polymers, and inorganic nanomaterials (Huang & Lovell, 2017; Tabish et al., 2020; Zhang et al., 2016; Zhu, Zhang, et al., 2019). Besides, different sources have been explored to excite these nanomaterials encompassing UV–vis, NIR, and second window NIR‐II light, enabling a wide range of clinical applications (Chen & Zhao, 2018). Due to the high energy for intense fluorescence, UV–vis light has been widely studied as the most conventional irradiation for phototriggered systems. Given the significant advances in this realm, this section highlights the progress of UV–vis light‐triggered theranostic NGs made in the past few years, focusing on two different systems, polymer NGs, and hybrid organic–inorganic NGs. Table 2 includes some recent examples relating to UV–vis light as the irradiation. Nanosystems that sensitively respond in the short‐wavelength UV window (<400 nm) were the first platform applied in clinical theranostics in the 1970s, and since then, the field has expanded broadly (Xiong et al., 2020; Zhang et al., 2016). One of the most typical approaches relies on endowing nanomaterials with photosensitive moieties such as coumarin, *o*‐nitrobenzyl, and disulfide derivatives that undergo irreversible bond cleavage upon UV irradiation (Ai et al., 2016; Stefanello et al., 2017). These functional groups may be presented as labile linkers between the nanoprobe and the therapeutics (Kohman et al., 2016), units within building blocks of nanoprobes (Huang et al., 2016), or a protective “photocage” (Tan et al., 2015). For instance, Meng et al. designed UV‐responsive degradable NGs from hyaluronic acid‐*g*‐7‐*N*,*N*‐diethylamino‐4‐hydroxymethylcoumarin (HA‐CM) for remotely controlled intracellular delivery of fluorescent DOX (Hang et al., 2017). As schematized in Figure 6a, after UV irradiation (315–400 nm) for 5 min, cleavage of urethane bonds that connect coumarin derivative to HA degraded the NGs. It activated the DOX release in CD44‐positive MCF‐7 cells, allowing cellular tracking by FLI. In another example, Tan et al. developed a DNA aptamer‐hyperbranched polymer (HBP)‐based NG containing pendent *o*‐nitrobenzyl moieties in its structure (Yang et al., 2018). DOX was loaded in the core of these self‐assembled constructs. Under UV irradiation (365 nm), the *o*‐nitrobenzyl moieties were rapidly cleaved from the polymer pendant chains, inducing the disassembly of the nanocarriers and the release of the payload. Then, the cellular localization of DOX could be followed by FLI.

**TABLE 2:** Summary of recently reported NGs for phototriggered theranostics

Nanogels composition	Imaging techniques	Therapeutics	Imaging agents	Refs.
HA	FLI	DOX	Fluorescent drug	Hang et al. (2017)
DNA‐*g*‐hyperbranched polymer	FLI	DOX	Fluorescent drug	Yang et al. (2018)
5‐Aminolevulinic acid‐s‐DOX	FLI	DOX/PDT (Protoporphyrin IX, PpIX)	Fluorescent drug/dye	Wang, Zu, et al. (2020)
dPG	FLI	DOX	Fluorescent dye/peptide	Nagel et al. (2020)
PpIX‐1‐methyltryptophan (1MT)	FLI	PDT (PpIX)/immunotherapy (1MT)	Fluorescent dye	Song et al. (2018)
PNIPAM‐dPG	PTI	PTT (polyaniline, PANI)	Semiconductive polymers	Molina et al. (2016)
γ‐PGA	PAI	PTT (PANI)	Semiconductive polymers	Zhou et al. (2017)
		PTT (polypyrrole, PPY)/RT	Semiconductive polymers	Zhou et al. (2018)
PNIPAM/PNIPMAM	PAI/PTI	PTT (PPY)/methotrexate	Semiconductive polymers	Theune et al. (2019)
PNIPAM‐dPG	PTI	PTT (PHMeEDOT)/diclofenac	Semiconductive polymers	Puiggalí‐Jou et al. (2021)
Poly(NIPAM‐*co*‐acrylic acid)	PAI/FLI	PTT (PDA)/DOX	Semiconducting polymer/fluorescent drug	Pu et al. (2021)
DNA‐*g*‐polycaprolactone	PTI/FLI	PTT (PDA)/siHsp70‐Cy5.5	Semiconducting polymer/fluorescent dye	Ding et al. (2020)
Cell membrane@gelatin	PAI/PTI/FLI	PTT (MB)/cisplatin	Fluorescent dye	Zhai et al. (2018)
Ag_2_S QDs@ polypeptides	PAI/FLI	PTT (Ag_2_S QDs)	Novel metal nanoparticle	Zhao et al. (2018)
Carbon dots (CDs) @ Chitosan (CH)	PTI/FLI	PTT (CDs)/DOX	Carbon nanoparticle/fluorescent drug	Wang et al. (2017)
Upconverting nanoparticles@CH	FLI	FITC‐BSA	Fluorescent protein	Jalani et al. (2016)
Au@HA	FLI	DOX	Metallic nanoparticle/fluorescent drug	Lin et al. (2021)

Since UV light shows some limitations, like potential photodamage to living tissues and poor tissue penetration depth, the approach of photolabile linkages has also been expanded to visible light‐responsive organic moieties, such as vitamin B12 derivatives (Shell & Lawrence, 2015) or trithiocarbonates (Kang et al., 2021). However, nanocarriers containing organic dyes absorbing in the visible range, like porphyrins (Yap et al., 2020; Zhang & Lovell, 2012), are among the most popular platforms for visible light‐mediated theranostics. An interesting example was recently reported by Xu et al., where a GSH‐responsive multifunctional NG, dubbed DSA, was prepared by chemical cross‐linking of DOX and 5‐aminolevulinic acid (ALA), a precursor of protoporphyrin IX (PpIX), with a disulfide linker (DBHD) (Wang, Zu, et al., 2020). As shown in Figure 6b, the biosynthesis of fluorescent PpIX in the mitochondria of tumor cells could serve as a real‐time imaging probe. Meanwhile, visible light‐irradiation of PpIX (660 nm) could convert oxygen into toxic reactive species for efficient PDT.

Another attractive strategy involving UV–vis light relies on the design of protease‐activated FLI nanotheranostic (Hou et al., 2015; Li et al., 2015). Here, Förster resonance energy transfer (FRET) is produced between a quencher and a UV–vis light‐absorbing chromophore covalently linked through a peptide substrate of proteases as caspases or matrix metalloproteinases (MMPs) (Zhang et al., 2018). Through this approach, Zhang et al. synthesized self‐assembled nanoparticles from a chimeric peptide, PpIX‐1MT, which combines fluorescent photosensitizer PpIX with an immune checkpoint inhibitor 1MT via the link of a caspase‐responsive peptide sequence, Asp–Glu–Val–Asp, for synergistic PDT and immunotherapy (Song et al., 2018). Upon visible light irradiation (630 nm), the PpIX‐1MT nanoparticles generated reactive oxygen species, inducing tumor cell apoptosis. Consequently, the expression of caspase‐3 and the generation of tumor antigens were promoted, triggering an intense immune response. Besides, the delivered 1MT after caspase‐mediated cleavage could strengthen the immune system activating CD8+ T cells effectively.

**FIGURE 7:**
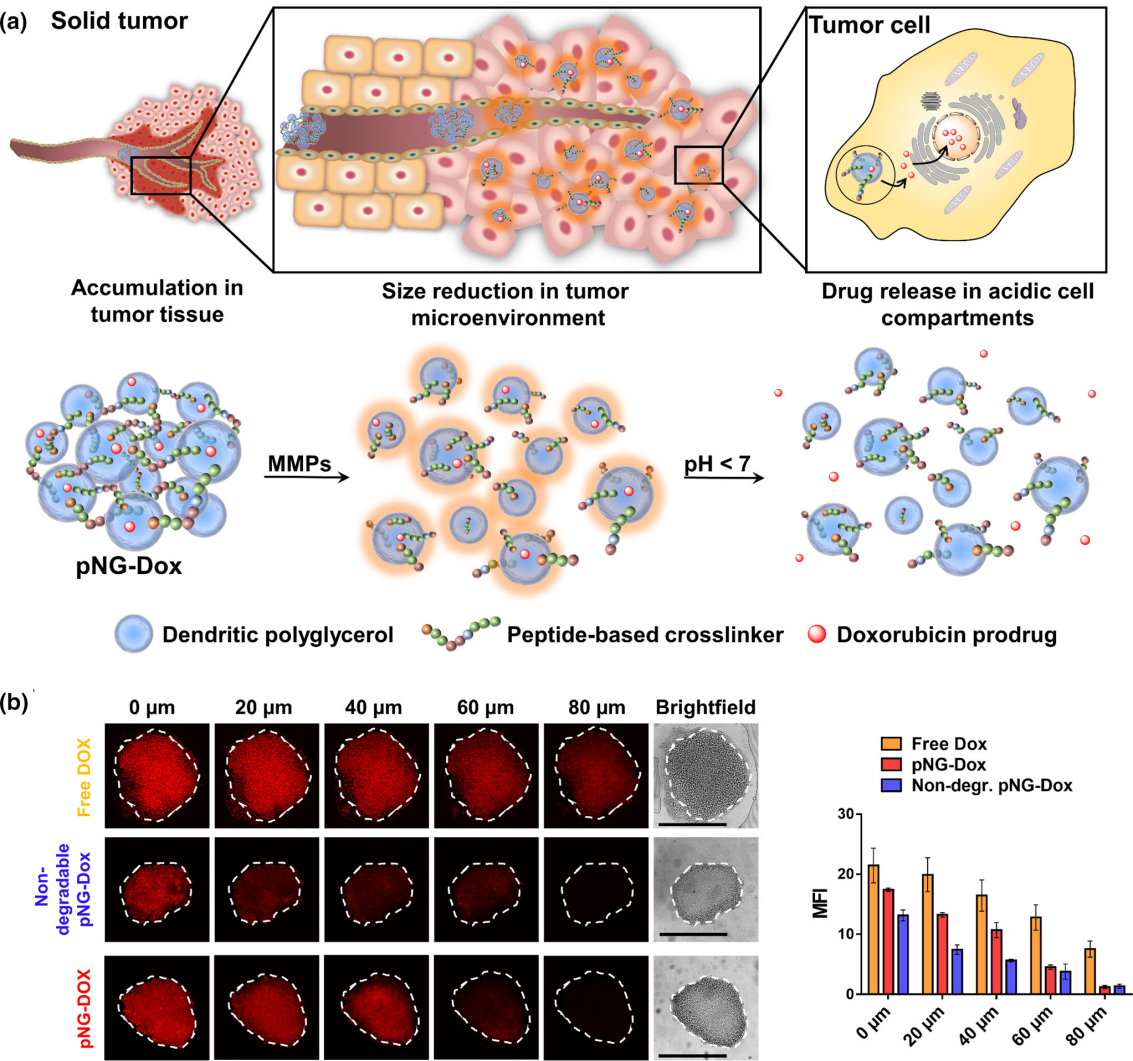
(a) Schematic representation of the mechanism proposed for the multistage drug delivery by pNG‐DOX. (b) Penetration of pNG‐DOX into MCTS: Comparison of free DOX, degradable multistage pNG‐dox, and the non‐degradable control (left) and the mean fluorescence intensity of DOX over the area of the MCTS for different penetration depth (right). Reprinted with permission from Nagel et al. (2020). Copyright 2020. Ivyspring International Publisher

Following this line, Calderón et al. recently reported the preparation of theranostics dendritic polyglycerol (dPG)‐based NGs, using an MMP‐7‐sensitive fluorogenic peptide as cross‐linker, bearing a dye pair that exhibits FRET (Nagel et al., 2020). Moreover, the chemotherapeutic drug DOX was conjugated through a pH‐sensitive linker to the dPG NGs, creating a multistage delivery system (pNG‐DOX). As shown in Figure 7a, in the first stage, pNGs‐DOX could be degraded by MMPs overexpressed in solid tumors. After the resulting size reduction, the remaining NG fragments easily penetrated into deeper areas of tumor tissue where the DOX release was favored due to the acidic pH in intracellular compartments. The authors investigated the penetration of these nanocarriers in multicellular tumor spheroids (MCTS). They demonstrated that DOX penetration from the degradable pNG‐DOX was notably increased compared to the non‐degradable control, especially for deep sections of the tumor spheroids (Figure 7b). These findings confirm that after pNGs degradation, the tiny NG fragments have a unique ability to penetrate deep regions of the 3D tumor model.

Although novel UV–vis responsive nanosystems have been prepared in the last few years, the full expansion of this theranostic field is still handicapped mainly due to potential DNA damage in UV irradiation and the low tissue penetration of both wavelengths range. However, both light sources are cheap and easily accessible; and therefore remain one of the most important alternatives in clinic theranostics, though often limited to treating skin‐associated diseases. With the development of more efficient and safer irradiation sources, like NIR light, the future of UV–vis theranostic is unclear, but certainly, it will be available in the clinics for the following years.

## 4 NANOTHERANOSTICS BASED ON NIR LIGHT AS TRIGGER

To overcome the shortcomings of UV–vis light related to high risk of tissue damage and low penetration, NIR‐light gradually turns into the most popular stimulus for phototriggered nanotheranostics at present, mainly due to its deeper tissue penetration with very low fluorescence interference from the tissue itself (Thangudu et al., 2021; Yan et al., 2021). Besides, its low energy induces less tissue damage than other excitation sources (Zhao et al., 2019). For these reasons, NIR‐responsive smart nanosystems have been at the forefront of the latest advances in cancer theranostics (Kim et al., 2016; Wang, Hou, et al., 2018). Table 2 shows some recent examples about theranostic NGs systems based on NIR‐light as irradiation.

**FIGURE 8:**
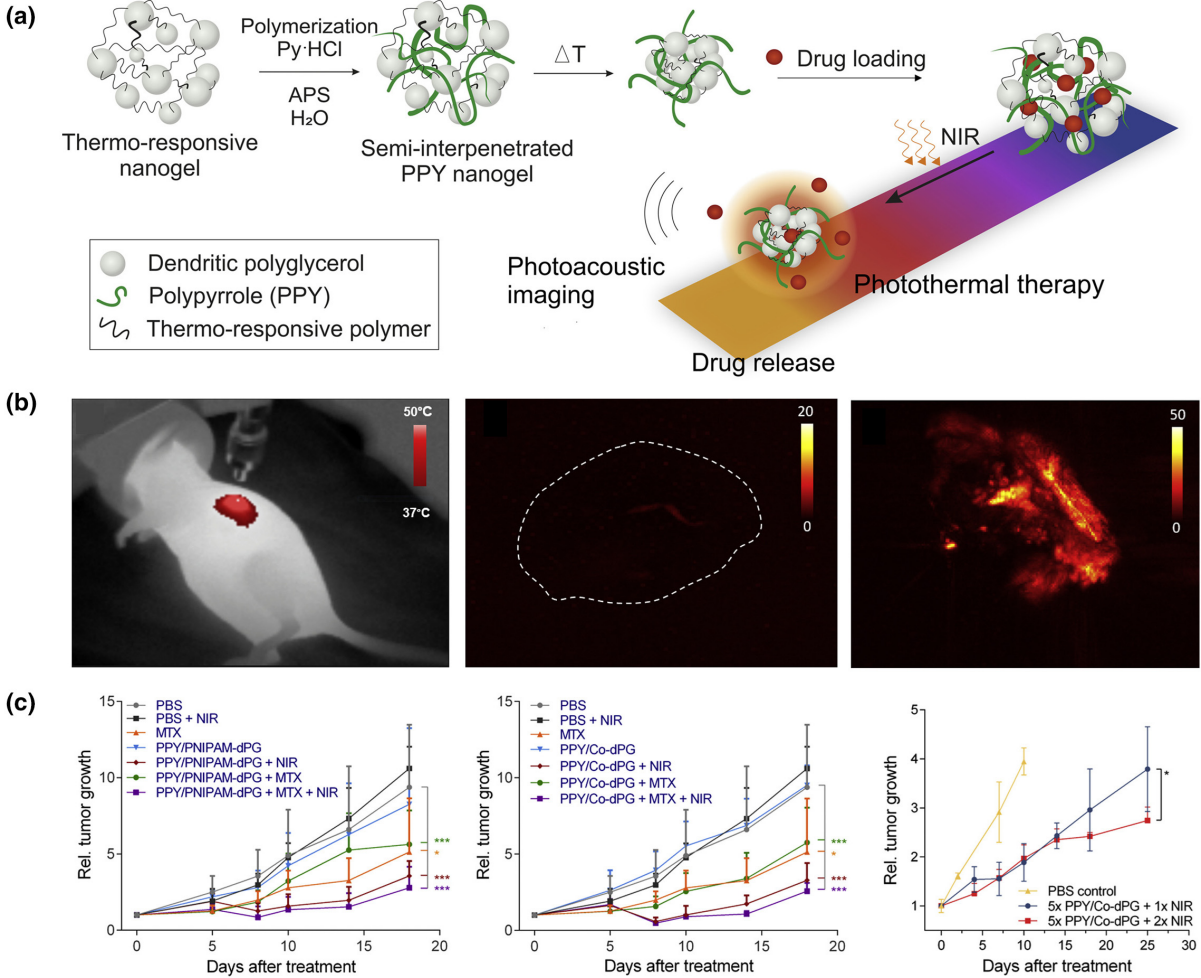
(a) Dual responsive NGs by semi‐interpenetration of PPY into thermo‐responsive NGs. (b) PTI of mice under NIR irradiation with i.t. injected NGs (left); PAI of an untreated control tumor (center) and tumor after injection of NGs followed by NIR irradiation ex vivo (right). (c) Rel. tumor growth over time of mice treated i.t. with MTX‐loaded PPY/PNIPAM‐dPG (left), and PPY/co‐dPG NGs, exposure to one (center) and multiple (right) NIR irradiation cycles. Reprinted with permission from Theune et al. (2019). Copyright 2019. Elsevier

In the past few years, an assortment of nanotheranostic platforms have been developed based on semiconducting polymers (Li et al., 2018; Li & Pu, 2020; Rejinold et al., 2021; Sarkar & Levi‐Polyachenko, 2020), organic dyes (Jung et al., 2018; Leitão et al., 2020; Lv et al., 2020; Xu, Li, et al., 2020), and inorganic materials like noble metal nanoparticles (Gao et al., 2021; Pohanka, 2020; Wang, Hou, et al., 2018; Wang, Wu, et al., 2020), carbon nanomaterials (Chung et al., 2021; Sajjadi et al., 2021; Saleem et al., 2018; Tade & Patil, 2020), and upconverting nanoparticles (UCNPs) (Le & Youn, 2020; Liang et al., 2020; Ovais et al., 2020; Zeng et al., 2017). Different semiconducting polymers have been explored for fabricating NIR‐sensitive NGs, including polyaniline (PANI) (Molina et al., 2016; Zhou et al., 2017), polypyrrole (PPY) (Theune et al., 2019; Zhou et al., 2018), poly(3,4‐etilendioxitiofeno) (PEDOT) (Cheng et al., 2012; Puiggalí‐Jou et al., 2021), and polydopamine (PDA) (Ding et al., 2020; Pu et al., 2021). In this field, Calderón and coworkers pioneered the development of semi‐interpenetrated polymer conducting NGs for cancer theranostic. For instance, they prepared thermoresponsive nanomaterials with strong NIR absorbance by semi‐interpenetrating PANI into dPG‐crosslinked PNIPAM‐based NGs (Molina et al., 2016). These polymeric nanocomposites proved to be highly efficient in inducing local hyperthermia for PTT in in vitro and in vivo studies. Following this approach, Shi et al. also reported the preparation of polyaniline‐loaded γ‐PGA NGs, cross‐linked with cystamine dihydrochloride (Cys), for PAI/PTT (Zhou et al., 2017). γ‐PGA/Cys@PANI NGs showed excellent light‐to‐heat transduction properties and biocompatibility, and were suitable for PAI‐guided PTT of both cancer cells in vitro and a xenografted tumor model in vivo. In a follow‐up study by this group, γ‐PGA/Cys NGs were semi‐interpenetrated with PPY for cooperative PTT and X‐ray radiation‐mediated radiotherapy (RT) for enhanced tumor theranostic (Zhou et al., 2018). The hybrid NGs possessed excellent photothermal conversion efficiency (64.7%) and stability upon NIR irradiation, and showed outstanding performance for PAI and PTT of cancer cells and tumor xenografts. In this line, the same group has recently reported on the design of PPY‐semi‐interpenetrated thermoresponsive NGs built from PNIPAM and poly(*N*‐isopropyl methacrylamide) (PNIPMAM), using dPG as cross‐linker (Figure 8a; Theune et al., 2019). As it can be seen in Figure 8b, these NGs showed excellent PTI and PAI ability. Besides, the anticancer drug methotrexate (MTX) was loaded into the NGs; hence they can serve as nanoprobes for combinational photothermal and chemotherapy. This synergistic approach showed efficient tumor growth inhibition for in vitro and in vivo models. The authors evaluated the relative tumor growth in mice models after dual treatments for different NGs compositions (Figure 8C). Even though appreciable differences were not observed when varying the NG type, the development of the tumor volume was considerably inhibited upon several NIR expositions during 25 days of treatment.

Although less explored until now, PEDOT derivatives have also been combined with NGs for controlled drug delivery in cancer theranostic. In a recent study by Puiggalí‐Jou, Alemán, Calderon et al., multitasking NGs from poly(hydroxymethyl 3,4‐ethylenedioxythiophene) (PHMeEDOT) and PNIPAM‐dPG were designed for combined mild PTT and chemotherapy (Puiggalí‐Jou et al., 2021). Diclofenac (DIC), a non‐steroidal anti‐inflammatory molecule exhibiting potent anticancer effects, was encapsulated within the NGs, and its release could be detected by electrochemical analysis thanks to the PHMeEDOT conductivity. These NGs showed excellent photothermal conversion and, in combination with DIC treatment, could kill 95% of cancer cells in vitro after 15 min NIR irradiation.

Unlike synthetic PANI, PPY, and PEDOT, PDA is a biobased semiconducting polymer obtained from the self‐polymerization of dopamine in an alkaline medium (Ryu et al., 2018). Since Prof. Lu's group reported their photothermal properties in 2012 (Liu et al., 2013), PDA has been broadly used in theranostic. In a very recent work, thermo‐/pH‐responsive poly(NIPAM‐*co*‐acrylic acid) (PNA) NGs were covered with a PDA layer to obtain multifunctional nanosystems for breast cancer therapy (Pu et al., 2021). The proposed nanoparticles were further conjugated with folic acid (FA) as a targeting agent for folate receptors and loaded with DOX. In vitro experiments demonstrated that PNA‐DOX@PDA‐FA nanoparticles could be phagocytized by 4T1 tumor cells, destroying them through synergetic chemo‐PTT. In another example, DNA‐grafted polycaprolactone (DNA‐*g*‐PCL) assembled NGs were coated with PDA as a therapeutic complex for siRNA‐mediated low‐temperature PTT (Ding et al., 2020). siRNAs targeting the heat‐shock‐protein 70 (siHsp70) served as a cross‐linker of DNA‐*g*‐PCL through nucleic acid hybridization. At the same time, the PDA layer protected the NGs against enzymatic degradation and endowed them with excellent NIR photothermal conversion. Furthermore, siHsp70 was labeled with Cy5.5 dye for FLI. Among organic dyes, heptamethine cyanines and porphyrins have been extensively studied, being currently indocyanine green (ICG) the only one approved by the FDA for clinical applications (Jung et al., 2018). In a recent example, a lipophilic NIR fluorescent cyanine dye (DIR) was introduced into glutathione‐sensitive NGs for FLI. DOX and mannose were co‐loaded to perform a combined chemo/immunotherapy. Specifically, DOX‐induced chemotherapy could cause tumor immunogenic cell death (ICD), manifesting the release of high‐mobility group box 1 (HMGB1) and the expose of calreticulin (CRT) on the cell membrane, resulting in an antitumor immune response to maximize the immune‐regulation effects from mannose (Ma, Yang, et al., 2021). Meanwhile, FIL was utilized to track the in vivo biodistribution and pharmacokinetics of the NGs. In another example, a syringeable immunomodulatory multidomain nanogel vesicle (iGels) was developed by Lim et al. (Song et al., 2019). iGels were labeled with NIR dye 800 for fluorescent tracking of the nanosystem retention. These vesicles, encapsulating gemcitabine and imiquimod, activated the recruitment of antigen‐presenting cells (APCs) and generated antigen‐specific T cells, which caused immunogenic cell death and the reshape of TME, preventing tumor metastasis and recurrence. It was worth noting that the corresponding fluorescent signal was detected even at the injection site after 2 weeks, compared with a few days for the injected free dyes. It could be explained as the NGs carriers decelerate the diffusion of the dye molecules leading to longer retention time and accumulative concentration.

Moreover, cyanine dyes have recently attracted much research attention due to their ability to self‐assemble into J‐aggregates, characterized by red‐shifted absorption, emission bands, and increased absorption coefficients. A compelling review article on the recent progress in NIR J‐aggregates for cancer theranostic has just been published by Zhang et al. (Xu et al., 2021).

**FIGURE 9:**
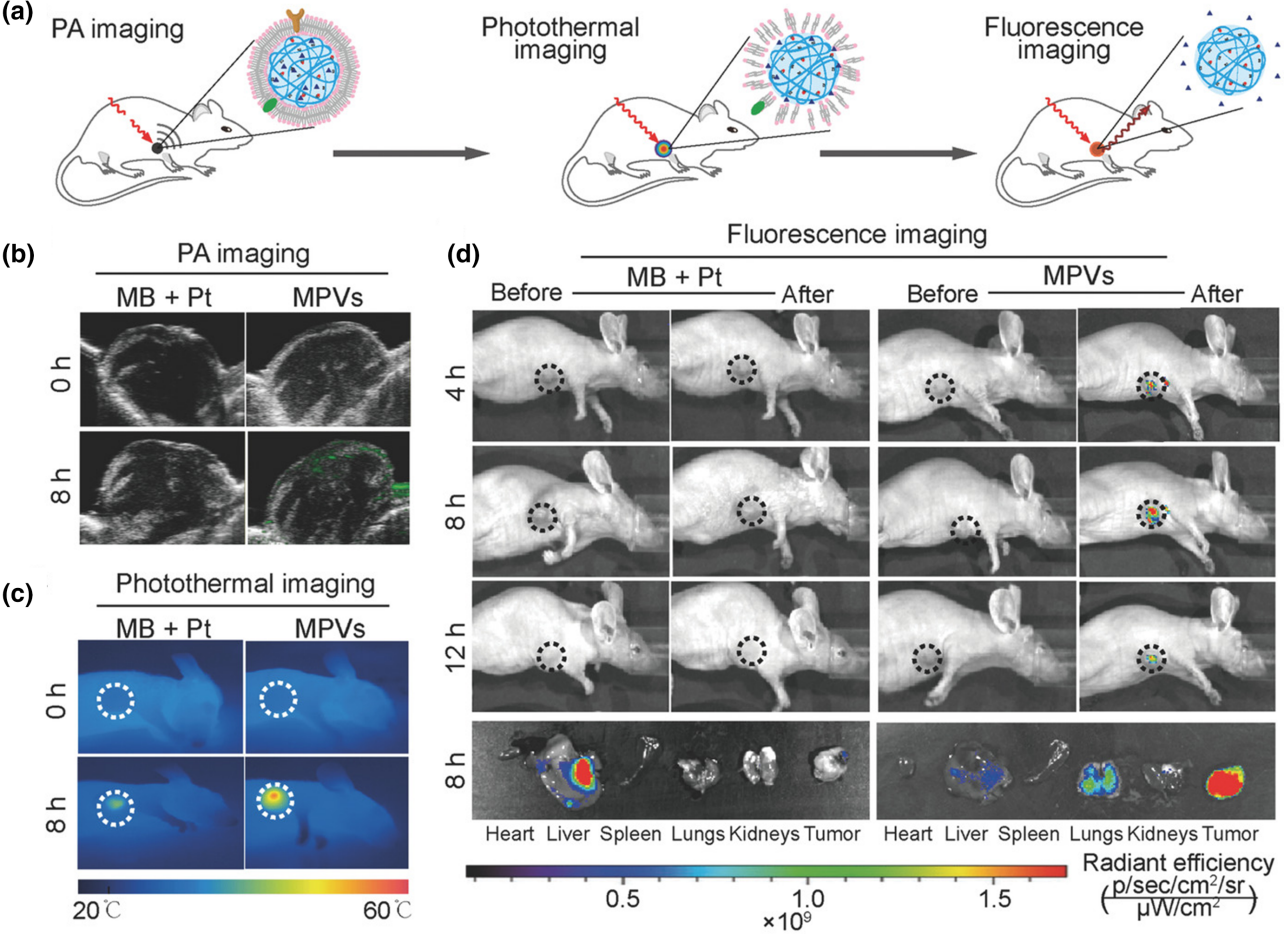
(a) Schematic illustration of the tracking of naive, being activated, and active MPVs in 4T1 tumor‐bearing mice using photoacoustic, photothermal, and fluorescence imaging. PA, PT, and FL imaging modalities of cell membrane‐coated gelatin NGs in 4T1 tumor‐bearing mice. (b) Photoacoustic imaging (PAI) of the intratumoral accumulation of naive MPVs at 0 and 8 h after intravenous administration. (c) The infrared thermographic images of mice treated with MPVs and localized irradiation (671 nm, 0.68 W/cm^2^, 4 min). (d) Representative fluorescence images of MPVs treated 4T1 tumor‐bearing mice and resected major organs and tumors after localized laser irradiation. In these experiments, a mixture of free drugs was used as a control. Reprinted with permission from Zhai et al. (2018). Copyright 2018. Wiley

Methylene blue (MB) is another important dye in theranostic, which was recently employed for preparing gelatin NGs armored with a cell membrane (CM); and co‐loaded with cisplatin drug (Zhai et al., 2018). As shown in Figure 9a, this interesting nanomaterial combines in only one platform PAI, PTI, and FLI with localized hyperthermia, PDT, and chemotherapy. This synergistic treatment effectively killed 4T1 breast cancer cells, resulting in primary tumor regression and 97% inhibition of pulmonary metastasis without significant toxicity in mice models. High photoacoustic contrast was observed in the tumors of mice after 8 h of i.v. NGs injection, while no signal was noticed in mice receiving free drugs (Figure 9b). Besides, the temperature of the tumor notably increased (maximum 56°C) when irradiating with NIR‐light (671 nm, 0.68 W/cm^2^, 4 min), enabling the imaging of the NGs by PTI (Figure 9c). Upon localized irradiation, strong fluorescence was detected in NGs‐treated mice, showing a maximum intensity 8 h after the treatment (Figure 9d). The main organs and tumors were analyzed, and the results confirmed that only the i.t. injected NGs were activated compared to free drugs administrated under the same conditions, which showed no significant fluorescence signals.

Inorganic nanoparticles represent a major group in NIR‐mediated theranostics, though their clinical use is still strongly questioned due to long‐term biosafety concerns (Kim et al., 2018). Under this scenario, hybrid inorganic–organic NGs appear as versatile platforms with improved biocompatibility for many advanced therapeutic applications. In this field, Liu et al. presented a novel multifunctional Ag_2_S quantum dots (QDs)‐polypeptide hybrid NG, which combines the capability of targeted NIR‐II FLI, PAI, and PTT for cancer theranostic (Zhao et al., 2018). They used genetically engineered polypeptides (PC_10_A and PC_10_ARGD) to form self‐assembled physical networks, where hydrophobic Ag_2_S QDs could be loaded. In vivo studies showed that the temperature of tumor sites treated with Ag_2_S‐QD@PC_10_ARGD reached around 61°C after 810 nm laser irradiation at a power density of 2.5 W/cm^2^ in 2 min, which was enough to ablate the tumor.

**FIGURE 10:**
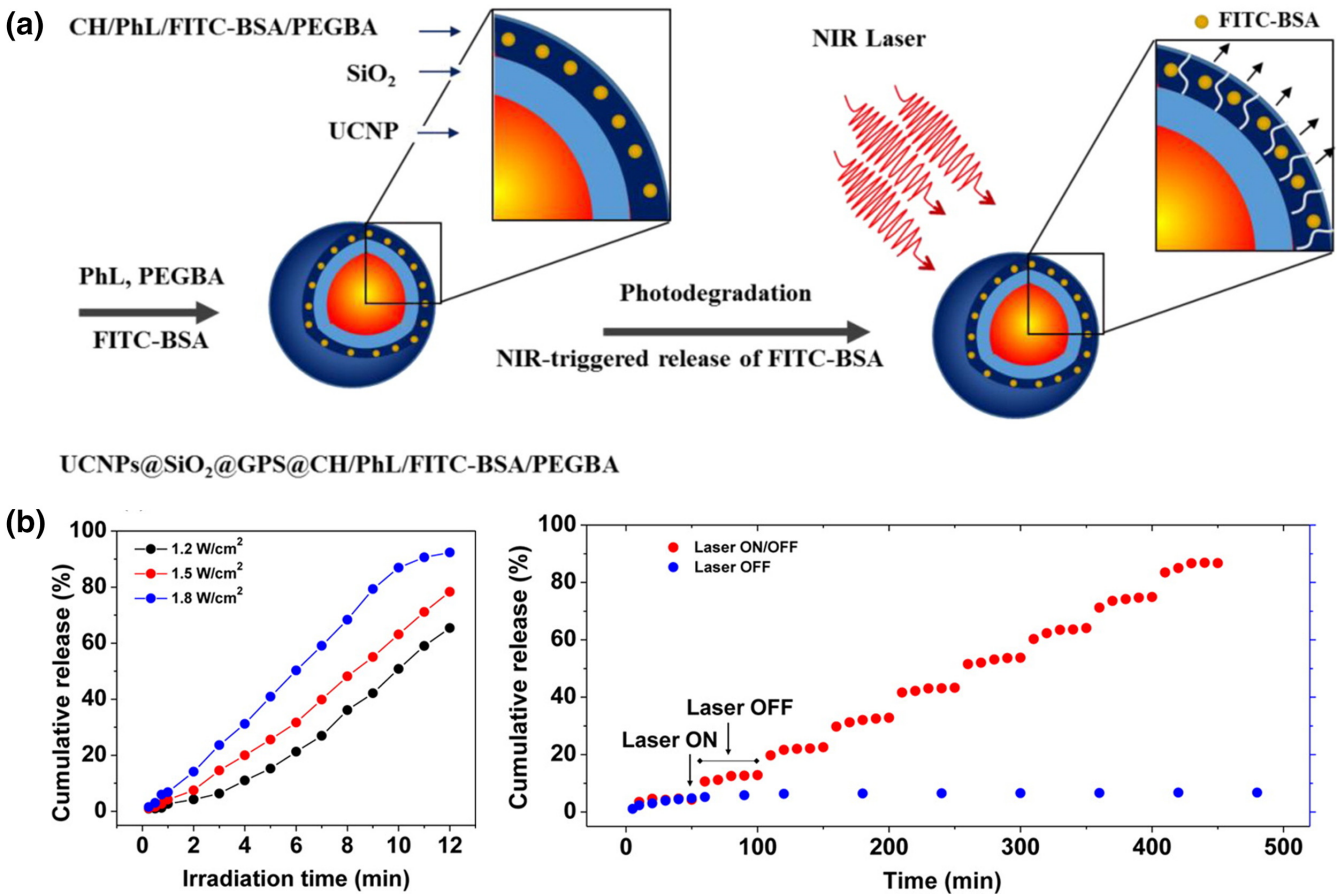
(a) Schematic illustration of the synthesis of photodegradable hydrogel‐coated UCNPs and drug release mechanism. (b) Cumulative FITC‐BSA release from the NGs as a function of irradiation time at different laser powers (left) and under NIR laser irradiation ON/OFF cycles (right).Reprinted with permission from Jalani et al. (2016). Copyright 2016. American Chemical Society

Biocompatible chitosan (CH)‐carbon dot (CD) hybrid NGs (CCHNs) for synergistic photothermal–chemotherapy were also reported by Zhou et al. (Wang et al., 2017). These NGs were prepared by a nonsolvent‐induced colloidal particle formation of chitosan–CD complexes assisted by ethylenediaminetetraacetic acid (EDTA) in water. DOX was encapsulated into the CCHNs through π–π stacking and electrostatic attractions with CD. In vivo tumor therapy of DOX‐loaded CCHNs resulted in almost total tumor growth inhibition after 18 days of treatment. In another exciting study, Cerruti et al. engineered NIR to UV–vis–NIR UCNPs consisting of LiYF_4_:Yb^3+/^Tm^3+^@SiO_2_ coated with a hydrogel layer of CH cross‐linked with a UV‐photocleavable cross‐linker (PhL), 2,5‐dioxopyrrolidin‐1‐yl (1‐(5‐methoxy‐2‐nitro‐4‐(4‐oxo‐4‐[pent‐4‐yn‐1 ylamino]butoxy) phenyl) ethyl) carbonate (Jalani et al., 2016). In addition, polyethylene glycol‐bisazide (PEGBA) was introduced to complete the cross‐linking by reacting the azide groups from PEGBA with the acetylene groups present on the free ends of PhL via click chemistry. Then, fluorescent‐bovine serum albumin (FITC‐BSA) was encapsulated inside the gel layer. As depicted in Figure 10a, under 980 nm excitation, the upconverted UV emission cleaved the PhL cross‐links and instantaneously released the fluorescent protein. The UCNP system showed excellent biocompatibility toward nucleus pulposus cells, and drug release can successfully be achieved as deep as 2 cm inside tissues, while the NIR emission band of UCNPs at 792 nm can be used to track particles under at least 1.5 cm thickness. Besides, the authors demonstrated that the irradiation power could control the cumulative release profile of FITC‐BSA, as shown in Figure 10b (left). Similarly, by performing on/off irradiation cycles, a sustained release with a maximum of around 80% could be attained (Figure 10b, right).

Moreover, gold nanoparticles@HA hybrid NGs were also recently developed (Lin et al., 2021). First, a vinyl group‐bearing HA derivative was cross‐linked with cystamine bisacrylamide (CBA) to obtain bioreducible NGs sensitive to intracellular GSH. Then, gold clusters were grown in the NGs by the in situ reduction of HAuCl_4_. These hybrid NGs (mHA‐GC) were loaded with DOX, and their tumor cell inhibition ability was evaluated in in vitro and in vivo models. Interestingly, the NGs showed to have excellent NIR FLI capacities, allowing the tumor visualization with good resolution. The authors found strong fluorescence signals in the liver at 1 and 2 h postinjection, indicating that a fraction of the hybrid NGs was rapidly cleared. All in all, the results showed that mHA‐GC, integrating bioimaging and microenvironment‐responsive drug‐release, had significantly superior anti‐tumor efficiency in H22 tumor‐bearing mice than free DOX.

Up to now, advanced photoactivated diagnosis has not been widely applied in clinics due to the limited tissue penetration ability of light and the unknown long‐term biotoxicity of photosensitizers. Nevertheless, the transition from academic research to clinics for the simplest systems has begun (Kolenc Peitl et al., 2019). Hopefully, more and more phototriggered theranostic will be applied in clinics in the near future.

## 5 MULTIMODAL THERANOSTICS

Nowadays, multimodal theranostic is one developing trend since it can simultaneously provide various treatments and diagnostic information from different perspectives to achieve enhanced efficacy and accuracy of diagnostic and/or therapeutic. It has been noted that multimodal imaging is beneficial for early tumor diagnosis, localization, and efficient therapy. Desirable properties, including high stability and biocompatibility, versatility for chemical modifications, excellent release capacity of payloads, and the prolonged blood circulation for CAs, prompt NGs to be ideal materials for designing the theranostic system with multimodalities. In this section, different approaches for functional NGs with multimodal imaging are discussed, namely: (1) combination of radiation and US imaging modalities; (2) combination of phototriggered imaging modalities; (3) combination of radiation, US and phototriggered imaging modalities.

**FIGURE 11:**
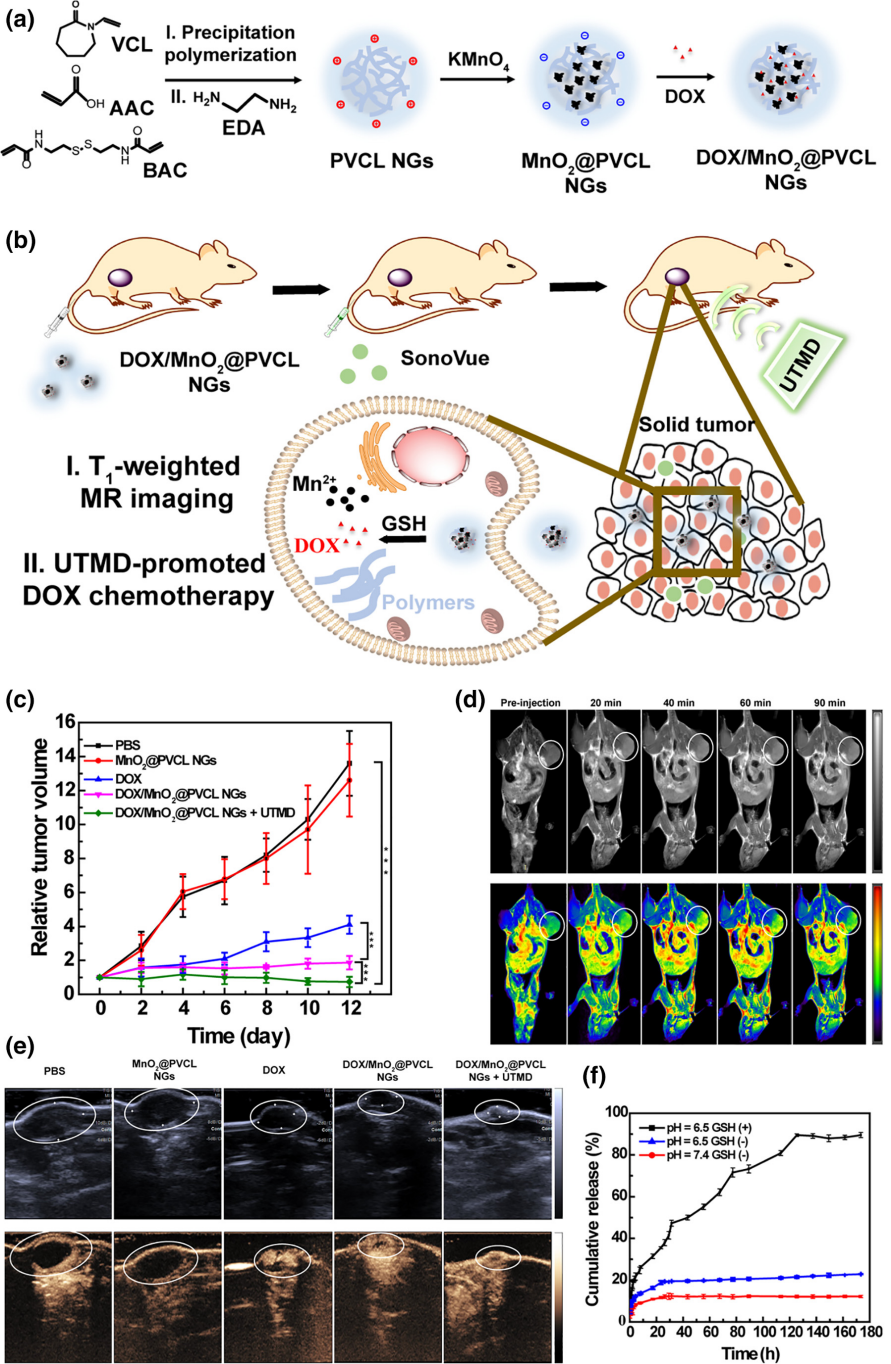
(a) Synthetic route of DOX/MnO2@PVCL NGs. (b) Schematic illustration of DOX/MnO2@PVCL NGs for UTMD‐promoted DOX chemotherapy and MRI. (c) DOX release profile from DOX/MnO_2_@PVCL NGs at pH 7.4 and 6.5 in the presence or absence of GSH (10 mM). (d) In vivo T_1_‐weighted MR imaging of subcutaneous B16 tumor‐bearing mice at different time points after the injection of DOX/MnO_2_@PVCL NGs. (e) B‐mode US images (upper) and CEUS imaging (below) of the tumor on day 12 after different treatments. (f) Relative tumor growth curve after different treatments. Reprinted with permission from Xu, Zhu, et al. (2020). Copyright 2020. Ivyspring International Publisher

Based on the radiation and US imaging modalities, MRI and US imaging are an exciting combination because of their non‐invasiveness for the body and high imaging speed. Through the advantages of the two imaging modalities, the cross‐sectional 3D information of soft tissue and the real‐time information can also be tracked (Wang et al., 2015). Recently, Shi and coworkers synthesized redox‐responsive poly(*N*‐vinyl caprolactam) (PVCL) NGs to load MnO_2_ and DOX for MRI and chemotherapy (Figure 11a,b; Xu, Zhu, et al., 2020). In this study, the ultrasound‐targeted microbubble destruction technique (UTMD), which has been proved to present no apparent effect for tumor growth, was applied to enhance the accumulation and cellular endocytosis of DOX and MnO_2_ specifically in the tumor site via sonoporation. Meanwhile, the ultrasound information was obtained as well. *N*,*N*′‐bis(acryloyl)cystamine (BAC), as a cross‐linker for NGs, was used for GSH‐triggered cleavage, based on the disulfide bond. Then, DOX and MnO_2_ were loaded by encapsulation and Mn–N coordination, respectively. Their cleavable ability boosted the release of DOX with the presence of GSH under pH 6.5 (Figure 11C). In vitro cellular uptake assay proved that the introduction of UTMD technique accelerated the cellular internalization of DOX. In vivo studies further demonstrated that this redox‐responsive hybrid NG system possessed favorable MR (Figure 11D) and US (Figure 11e) imaging performance and highly efficient chemotherapy (Figure 11f), upon the aid of UTMD technique. Additionally, the same group reported another study to integrate CT and MR imaging into a hybrid alginate/PEI‐Au‐Gd NG system, which can also be applied for PTT with the entrapment of Au NPs (Sun, Zhang, Zhang, Zhou, et al., 2018).

**FIGURE 12:**
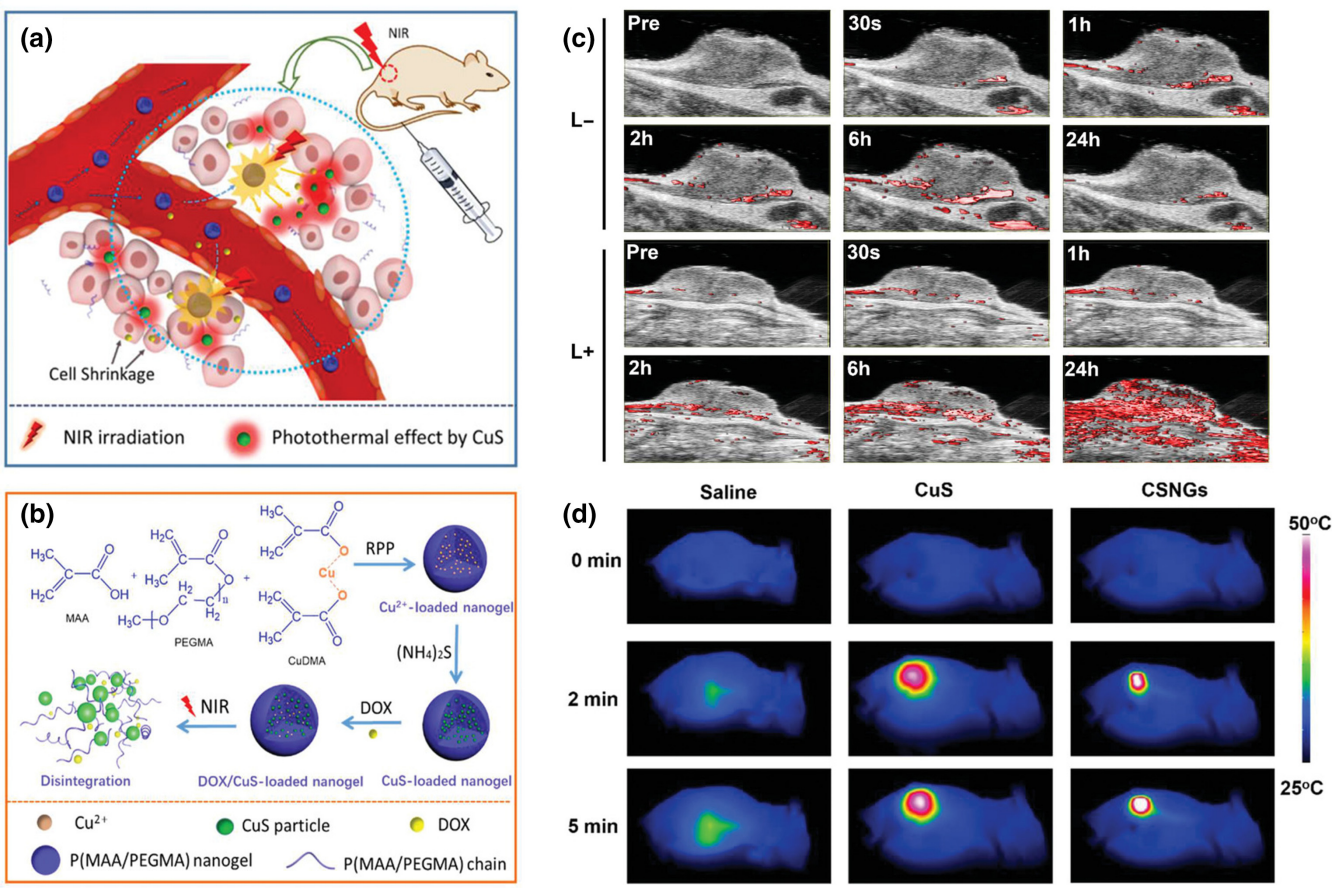
Schematic illustration of the theranostic mechanism (a) and the fabrication (b) of DOX/CuS‐loaded nanogels (DCSNGs). (c) In vivo PAI of mice bearing H22 tumor after the intravenous injection of DCSNGs, with or without laser irradiation at 2 h postinjection for 25 min. (d) Thermal images of tumor‐bearing mice with 5‐min irradiation of 808 nm NIR laser after intravenous injection with saline, CuS nanoparticles, and DCSNGs. Reprinted with permission from Zhang et al. (2019). Copyright 2019. Wiley‐VCH

Furthermore, different phototrigger imaging techniques can also be combined to establish a multimodal theranostic NG system, like the association of fluorescent and PAI modalities. PA imaging is the most popular candidate for multimodal theranostic, owing to its remarkable combination with phototrigger and acoustic imaging. For example, Wang et al. synthesized DOX‐encapsulated thermo‐destructible poly(methacrylic acid)‐*co*‐poly(ethylene glycol) methacrylate NGs with copper dimethacrylate as cross‐linker, applied for chemo/PT therapies and PA/FL imagings, as shown in Figure 12a,b (Zhang et al., 2019). Then, the loading of CuS was realized via the in situ growth by adding (NH_4_)_2_S. Under the irradiation of NIR light and acidic environment, NGs showed a robust in vitro DOX release, up to 87.3% in 6 h. In vivo PA imaging (Figure 12c) revealed that the NGs penetrated deeper into the tumor with the exposure of NIR laser. Furthermore, the FL imaging corroborated the good bio‐dispersion in the body and navigation ability toward tumors of the hybrid NGs. After being tracked by PAI, the temperature of the tumor site was recorded, showing a dramatic increase in 5 min under the irradiation of 808 nm‐laser (Figure 12d). It was illustrated that the photothermal effect could promote the combination of chemotherapy and PTT, and fluorescence and PAI, showing superior theranostic efficacy.

Taking advantage of radiated and phototriggered modalities, such as deep diagnosis, large imaging area, high accuracy, and spatiotemporal sensitivity, it is possible to design a NG platform with superior precision and efficiency for tumor theranostic. For instance, Cuggino, Calderón et al. have recently developed a dual‐modal imaging‐guided photothermal nanoplatform combining MRI and NIR‐induced FLI (Picchio et al., 2021). In this system, casein micelles were prepared using gadopentetic acid as the cross‐linker and MRI CA, while ICG J‐aggregates were stabilized into the hydrophobic pockets of the protein for efficient PTT and FLI. Interestingly, confined ICG J‐aggregates could act as monomer depots, allowing long‐term dye supply upon fast photobleaching by NIR light irradiation. Thus, this strategy allowed to achieve efficient multistep PTT for tumor theranostic. Besides, Shen et al. developed PEI NGs incorporating Gd and CuS molecules to integrate MR/PA imaging modality and PTT under the NIR irradiation. With the functionalization of FA, the NGs showed satisfied targeting dual‐modal imaging quality and PTT efficacy (Zhang et al., 2020).

Altogether, multimodal functional NG platforms help the development of systematic and precise cancer treatments. Integrating different imaging techniques, such as radiation, magnetic field, ultrasound wave, and light, and various treatments, such as chemotherapy, PTT, and PDT, will bring a bigger room for cancer nanotheranostic.

## 6 CONCLUSIONS AND PERSPECTIVES

Functional NGs have gained a great deal of attention in cancer nanotheranostics due to their superior features such as good biocompatibility, high capacity for transporting hydrophilic and hydrophobic payloads, and the opportunity for multifunctionalization. Considering the vast assortment of building blocks available for NGs design, a library of therapeutics platforms can be created offering unique flexibility over other types of nanosystems, like traditional liposomes or inorganic nanoparticles. For instance, stimuli‐sensitive units can be easily incorporated into the NGs structure affording smart delivery of cargoes and reaching successful therapeutic outcomes. Even more, functional monomers able to coordinate metal ions could be utilized to obtain nanoplatforms with imaging capacity for diagnosis. In this sense, since typical imaging agents are metal components or organic dyes, their combination with NGs can enhance their in‐vivo stability and residence time in the bloodstream, boosting imaging quality. In particular, due to the development of novel NGs that can efficiently stabilize innovative light‐sensitive CAs, phototriggered theranostic with a high temporal–spatial resolution has been dramatically impelled. Nevertheless, their limited tissue penetration leaves them much space for further investigation.

It should be noted that the transition from the academy to the clinic still remains to be the main challenge. Firstly, long‐term safety issues are rarely addressed in academic research. Although tens of biodegradable building blocks are commercially available for polymer synthesis, the fate of the theranostic NGs after in‐vivo applications should be appropriately evaluated. FDA and other agencies are willing to certify new reagents for clinical biomedicine, but the biotoxicity and immunotoxicity of novel nanosystems need to be identified, which takes a long time. Secondly, there is a huge lacune from the bench to the bedside regarding large‐scale production. Considering the several components and steps involved in synthesizing multifunctional nanotheranostics, tedious purification techniques are currently used in the laboratory, which are utterly unviable at the industrial stage and remain one of the major bottlenecks for product scale‐up. Lastly, due to low‐cost requirements for massive nanomedicine distribution, pharmaceutical companies tend to expand the market of already‐approved traditional nanomedicines instead of investing in expensive novel theranostic formulations.

Although there is a long way to go until clinical translation, new innovative theranostic NGs dealing with almost unexplored areas in the biomedical cancer space will be welcome in the future. Specifically, the nose‐to‐brain delivery of nanotheranostic to treat glioblastoma and neurodegenerative diseases has been barely investigated in the literature. The multimodal imaging ability of these nanosystems could be used to track the NGs in different brain areas, while they could be engineered for direct transport to the central nervous system. Other advanced technologies in combination with NGs, like CRISPR/CAS, also remain unexplored. Overall, NGs are promising nanomaterials for cancer theranostics in the nanomedicine field and will stand on higher steps in the future. It is hoped that this summary and overview of the current academic stage of tumor nanotheranostic will shed light on researchers to study novel functional NGs for biomedical applications.

## CONFLICT OF INTEREST

The authors have declared no conflicts of interest for this article.

## AUTHOR CONTRIBUTIONS

**Huiyi Wang:** Conceptualization (equal); formal analysis (equal); writing – original draft (lead). **Matias Picchio:** Conceptualization (equal); formal analysis (equal); writing – original draft (supporting); writing – review and editing (equal). **Marcelo Calderón:** Conceptualization (equal); funding acquisition (lead); project administration (lead); resources (lead); writing – review and editing (equal).

## RELATED WIRES ARTICLES

Nanogels for delivery, imaging and therapy

## Data Availability

Data sharing is not applicable to this article as no new data were created or analyzed in this study.
